# Unveiling the inhibitory effects of tannic acid and doxorubicin combination on pyruvate kinase M2 in breast cancer cells

**DOI:** 10.3389/fphar.2026.1818890

**Published:** 2026-07-16

**Authors:** Yi Wang, Ali El-Far, Ahmed A. Warda, Shymaa A. Mohamed, Mazaher Maghsoudloo, P. K. Hashim, Yaser H. A. Elewa, Alaa Elmetwalli, Soad K. Al Jaouni, Wenhong Zhao

**Affiliations:** 1 The First Affiliated Hospital of Jinzhou Medical University, Jinzhou, Liaoning, China; 2 Key Laboratory of Epigenetics and Oncology, The Research Center for Preclinical Medicine, Southwest Medical University, Luzhou, China; 3 Chemistry Department, Faculty of Science, Suez Canal University, Ismailia, Egypt; 4 Molecular Biology Unit, Medical Technology Centre, Medical Research Institute, Alexandria University, Alexandria, Egypt; 5 Research Institute for Electronic Science, Hokkaido University, Sapporo, Japan; 6 Department of Histology, Faculty of Veterinary Medicine, Zagazig University, Egypt; 7 Faculty of Veterinary Medicine, Hokkaido University, Hokkaido, Japan; 8 Prince Fahad Bin Sultan Chair for Biomedical Research, University of Tabuk, Tabuk, Saudi Arabia; 9 Department of Hematology/Pediatric Oncology, Yousef Abdulatif Jameel Scientific Chair of Prophetic Medicine Application, Faculty of Medicine, King Abdulaziz University, Jeddah, Saudi Arabia

**Keywords:** combination therapy, doxorubicin, glucose metabolism, PKM2, tannic acid

## Abstract

Breast cancer (BC) is the most commonly diagnosed cancer and the leading cause of cancer deaths among women worldwide. This study investigates the combined effects of tannic acid (TA) and doxorubicin (DOX) on glucose metabolism in estrogen receptor-positive (MCF7) and triple-negative (MDA-MB-231) BC cells by targeting pyruvate kinase M2 (PKM2). Cells were treated with TA, DOX, and their combinations, then analyzed for cytotoxicity, apoptosis, cell cycle progression, migration, and colony formation. PKM2 expression levels and activity were also measured. Results demonstrated that TA, alone and with DOX, effectively induced apoptosis and inhibited migration and colony formation in both cell lines. Notably, PKM2 mRNA expression was significantly reduced, particularly in TA-treated groups. Furthermore, glucose levels in the medium increased significantly following TA treatment, indicating reduced glucose uptake by cancer cells, and indicating that combining TA with DOX can disrupt glucose metabolism and diminish aggressive behaviors in BC cells. By targeting the glycolytic enzyme PKM2, TA lowers its expression, restricts glucose uptake, and enhances chemotherapy-driven apoptosis while limiting migration and colony formation. These findings underscore the potential of metabolic reprogramming through combining TA with DOX as a promising therapeutic strategy to improve BC treatment outcomes.

## 1 Introduction

According to the International Agency for Research on Cancer (IARC), the number of new cancer cases in 2022 reached almost 20 million (Bray et al., 2024). Breast cancer (BC) is the most commonly identified cancer and is the leading cause of cancer deaths among women around the world (Sedeta et al., 2023). BC can be categorized into molecular subtypes according to mRNA expression levels. These subtypes include luminal A, luminal B, human epidermal growth factor receptor 2 (HER2)-enriched, and triple-negative breast cancer (TNBC). The classification is based on the expression of estrogen receptors, progesterone receptors, and the overexpression of HER2 (Burguin et al., 2021; Łukasiewicz et al., 2021).

Glycolysis is the primary energy pathway driving cancer growth and metastasis. This metabolic shift from mitochondrial oxidative phosphorylation to aerobic glycolysis is referred to as the Warburg effect (Liberti and Locasale, 2016). The pyruvate kinase (*PKM*) gene encodes two variants, *PKM1* and *PKM2*, which are involved in glycolysis. PKM2 is an important enzyme involved in glycolysis found at higher levels in breast, prostate, lung, colon, and liver cancers. Its expression is enhanced by c-Myc, which is found in estrogen receptor-positive and TNBC subtypes (Yu et al., 2020).

It is important to note that PKM2 regulates the final rate-limiting step of glycolysis as a “metabolic gatekeeper”, and thus it is crucial to understand the relationship between its activity and glucose uptake (Wu et al., 2024). Normally, the enzyme PKM2 converts phosphoenolpyruvate to pyruvate, producing adenosine triphosphate (ATP). The glycolytic flux slows down when PKM2 is inhibited, resulting in the build-up of upstream glycolytic intermediates (Zahra et al., 2020; El-Far et al., 2022). This accumulation results in a feedback mechanism that downregulates the uptake of glucose either through the inhibition of hexokinase and phosphofructokinase or through the downregulation of the glucose transporter GLUT1 by transcription (Christofk et al., 2008; Benzarti et al., 2024; Liu et al., 2025). This means that inhibition of PKM2 leads to reduced glucose utilization, and it can be measured by higher levels of glucose in the culture media after the treatment.

Chemotherapy for BC involves various cytotoxic drugs, including alkylating agents, antimetabolites, and tubulin inhibitors (Nabholtz and Gligorov, 2005). Anthracyclines, such as doxorubicin (DOX), daunorubicin, epirubicin, and idarubicin, function by binding to DNA and hindering the macromolecular biosynthesis associated with BC (Gewirtz, 1999). Chemotherapeutics can cause significant side effects, and patients may become resistant to these medications. As a result, it is essential to seek a better understanding of new treatments that are more targeted and effective in addressing BC (Burguin et al., 2021).

A growing number of studies are investigating natural bioactive compounds to identify new and more effective treatment approaches for BC, whether used independently or alongside chemotherapeutics (El-Far, 2015; Li et al., 2017; Mitra and Dash, 2018; El-Far et al., 2020; 2021a; 2021b). Numerous studies have explored the use of natural bioactive compounds in combination with chemotherapeutic drugs, focusing on boosting the effectiveness of these treatments and reducing their side effects (El-Far et al., 2021b). Our previous comprehensive review has discussed the PKM2 inhibitors from natural bioactive compounds that are promising drug candidates for cancer therapy (El-Far et al., 2022).

Tannic acid (TA) is a mixture of gallic acid esters derived from glucose, present in various sources such as plants, fruits, teas, nuts, and coffee grains (Chen and Chung, 2000; Arapitsas, 2012). TA resulted in cell cycle arrest and apoptosis while reducing multiple cancer cell lines’ growth and migration rates (Ghasemian et al., 2023). TA is a natural bioactive substance that encourages programmed cell death and inhibits multiple forms of cancer (Kim et al., 2019; Kleszcz et al., 2025). The current study investigates the anticancer effects of TA, particularly in combination with DOX. It examines how these substances affect PKM2 activity in BC cells, offering potential new approaches for treating this aggressive cancer. The research is novel in its exploration of the combined impact of TA and DOX on PKM2, leading to BC cell apoptosis and alterations in glucose metabolism.

## 2 Materials and methods

### 2.1 Reagents and cell culture

Human BC cell lines MCF7 and MDA-MB-231 were sourced from ATCC (Manassas, VA, USA). High-glucose Dulbecco’s Modified Eagle Medium (DMEM, D0822), fetal bovine serum (FBS, F9665), penicillin/streptomycin (P4458), DOX (D1515), and TA (403040) were purchased from Sigma-Aldrich (MA, United States). Cells were maintained in high-glucose DMEM, enriched with 10% FBS, and supplemented with 1% penicillin/streptomycin.

### 2.2 Bioinformatic analyses

#### 2.2.1 Gene expression analysis of PKM

Gene expression data for BC samples were retrieved from the Cancer Genome Atlas (TCGA) database using the *TCGAplot* R package (Liao and Wang, 2023). Additionally, clinical data for all samples were downloaded from TCGA to facilitate further analyses. The gene expression data were normalized to transcripts per million (TPM), and expression levels were evaluated across different sample types, including normal, primary tumor, and metastatic BC tissues. Furthermore, we compared gene expression levels among various molecular subtypes of BC, including luminal A, luminal B, HER2-enriched, and basal. In addition to these analyses, we performed diagnostic evaluations by constructing receiver operating characteristic (ROC) curves to assess the ability of *PKM* to distinguish between BC subtypes and between normal and cancerous tissues. Prognosis-related analyses and survival analyses were conducted using Kaplan-Meier curves and Cox proportional hazards models to explore the association between *PKM* and patient outcomes, including overall survival.

#### 2.2.2 Protein-protein interaction (PPI) network

The protein-protein interactions (PPI) of *PKM* with other proteins were extracted from the STRING database (https://string-db.org/). For data extraction, experiments and databases were selected as active interaction sources, and a minimum required interaction score of 0.7 was used to ensure high-confidence interactions. Following the PPI network construction, we performed correlation analysis for all proteins involved in the network using BC expression data (Abutalebi et al., 2024). This allowed us to identify potential co-regulation patterns and functional associations between *PKM* and its interacting partners, providing deeper insights into their biological roles.

#### 2.2.3 Enrichment analysis

One effective bioinformatics technique for identifying biological patterns or pathways that are overrepresented in a particular gene or protein set is enrichment analysis, which provides insight into the putative functional roles of the genes or proteins. Gene set enrichment analysis was conducted using the *TCGAbiolinks* R package, which uses the Gene Ontology (GO) and Kyoto Encyclopedia of Genes and Genomes (KEGG) databases to determine whether biological processes, molecular functions, or signaling pathways are statistically enriched.

#### 2.2.4 Mutational analysis

The BC Mutation Annotation Format (MAF) file from TCGA was extracted using the *TCGAbiolinks* R package. The median *PKM* expression across all BC tumor samples was calculated, and the samples were then categorized into two groups, high-*PKM* and low-*PKM*, based on median. The median was chosen as the cutoff because it is a robust, unbiased statistical measure that divides the dataset into two equally sized groups, minimizing the influence of outliers compared to other approaches, such as mean-based or quartile-based splits (Györffy et al., 2010). This median-based stratification is a well-established method commonly used in TCGA-based BC expression studies.

Samples with expression levels lower than the median were placed in the low-*PKM* group, while those with expression levels higher than the median were placed in the high-*PKM* group. Using this approach, 557 samples were classified into the high-*PKM* group and 556 samples into the low-*PKM* group, ensuring sufficient statistical power in both groups for downstream analyses. Furthermore, the tumor mutational burden (TMB) score was calculated and analyzed to explore the relationship with *PKM* expression and investigate the heterogeneity of BC (Abutalebi et al., 2024).

### 2.3 Molecular docking assessment

The three-dimensional (3D) structure of the human PKM2 protein (ID: 5X1W) was obtained from the RCSB Protein Data Bank (RCSB PDB; https://www.rcsb.org/). The structures of TA and DOX were retrieved from the PubChem database (https://pubchem.ncbi.nlm.nih.gov/) in SDF format. The 2D SDF format of TA was converted to its 3D SDF format using Open Babel software. Molecular docking between the protein and ligands was performed using PyRx software (Dallakyan and Olson, 2015). The molecular interactions were analyzed using the PLIP server (Salentin et al., 2015) and visualized using PyMOL (http://www.pymol.org/pymol) software.

### 2.4 Molecular dynamics (MD) simulations assessment

Molecular dynamics (MD) simulations of the PKM2–ligands (TA or DOX) complexes were performed using GROMACS 2025.3 (Páll et al., 2020) in a periodic cubic box with the AMBER14SB force field (Maier et al., 2015) and TIP3P water. Ligand partial charges and optimized geometry were obtained from QM calculations using ORCA 6.0 (Neese, 2012; 2018) in two stages: gas-phase geometry optimization with B97-3c DFT, followed by single-point energy calculations using B3LYP-D3/def2-TZVP with the RIJCOSX approximation. RESP charges were derived from the ESP using the Multiwfn program. The ligand topology was generated with Sobtop software (http://sobereva.com/soft/Sobtop/), and ions were added to 150 mM NaCl. Energy minimization (500 steps conjugate gradient, tolerance 100.0 kJ/mol nm, step 0.01 nm) was followed by a 100 ps restrained run (1 fs step) and a 100 ns production run (2 fs step). Temperature (298.15 K) and pressure (1.0 bar) were controlled by V-rescale (τ = 0.2 ps) and C-rescale (τ = 0.5/2.0 ps), respectively. LINCS constrained bonds, PME handled long-range electrostatics (cutoff 1.0 nm), and Van der Waals used a Verlet cutoff (1.0 nm) with dispersion corrections. Center-of-mass motion was removed; angular-momentum removal and independent-temperature coupling were applied to the complex during production. Analyses included backbone root mean square deviation (RMSD), radius of gyration (Rg), root mean square fluctuation (RMSF), solvent accessible surface (SASA), and protein–ligand H-bonds. The binding free energy (ΔG_bind_) was calculated using MM-PBSA, which decomposes ΔG_bind_ into MM energy (ΔE_vdW_ and ΔE_ele_) and solvation components (ΔG_PB_ and ΔG_SA_). Although less accurate than FEP/TI, MM-PBSA is computationally efficient for ranking binders and analyzing molecular recognition. Per-residue decomposition identified hot-spot residues by quantifying each residue’s van der Waals, electrostatic, and solvation contributions to binding. ESP analysis was also performed to visualize charge distribution. Furthermore, 2D and 3D free energy landscapes (FEL) were constructed using Rg and RMSD as coordinates to map conformational stability. The dark blue low-lying region indicates the global free energy minimum, corresponding to the most thermodynamically stable conformation.

### 2.5 MTT [3-(4,5-dimethylthiazol-2-yl)-2,5-diphenyltetrazolium bromide] assay

To determine the half-maximal inhibitory concentration (IC50) values for TA and DOX, approximately 1 × 10^4^ MCF7 or MDA-MB-231 cells were seeded in 96-well polystyrene-coated plates. The cells were incubated for 24 h at 37 °C in a 5% CO_2_ environment. After incubation, the cells were exposed to different concentrations of TA (ranging from 0 to 400 μM) and DOX (ranging from 0 to 100 μM). Following an additional 24 h of incubation, the medium was removed, and MTT reagent (1.25 mg/mL) was added to each well. After a 2-h incubation period, formazan crystals formed, which were then dissolved in 100 μL of DMSO. The optical density of each well was subsequently measured at 570 nm using a microplate reader. *n* = 3 independent experiments.

Furthermore, in 24-well plates, we treated each well containing 5 × 10^4^ MCF7 or MDA-MB-231 cells with concentrations corresponding to the 0.50 IC50 values. We measured cell viability percentages using the MTT assay, comparing the results from treated wells to those of untreated control wells to determine the extent of cell cytotoxicity. The 0.5 × IC50 concentrations used for treatment were as follows: TA at 98.3 μM for MCF7 and 103.15 μM for MDA-MB-231, and DOX at 12.5 μM for both cell lines. These concentrations were used in all the following assays.

### 2.6 Annexin-V assay

Apoptosis analysis in treated MCF7 and MDA-MB-231 cells was conducted using flow cytometry, employing an annexin V-FITC and propidium iodide (PI) detection kit from BD Biosciences (San Jose, CA, USA). Fluorescence intensity was assessed using a FACSCalibur™ instrument (Becton Dickinson) and analyzed with CellQuest software. The gating strategy was as follows: forward scatter vs. side scatter was first used to exclude debris and doublets. Then, annexin V-FITC (FL1) and PI (FL2) fluorescence were plotted. Quadrant boundaries were set into lower left (annexin V^−^/PI^−^) = live cells; lower right (annexin V^+^/PI^−^) = early apoptotic; upper right (annexin V^+^/PI^+^) = late apoptotic; upper left (annexin V^−^/PI^+^) = necrotic/dead cells.

### 2.7 Cell cycle analysis

MCF7 or MDA-MB-231 cells were cultured in 6-well plates with 20 × 10^4^ cells until they reached full confluence. Subsequently, the cells were treated with TA, DOX, or their combinations. After 24 h, the cells were harvested, washed with PBS, and suspended in 70% ethanol. The cell cycle status of treated MCF7 and MDA-MB-231 cells was evaluated using a FACSCalibur flow cytometer (Becton Dickinson) and CellQuest software.

### 2.8 Scratch wound healing assay

MCF7 and MDA-MB-231 cell lines were grown in 12-well plates at a density of 7 × 10^4^ cells per well, with each well containing 1.5 mL of culture medium. Once the cells reached full confluence, a strip was created in the monolayer using a 200 μL pipette tip. Following this, the cells were rinsed twice with phosphate-buffered saline (PBS) and subsequently treated for 24 h with TA (98.3 μM for MCF7 and 103.15 μM for MDA-MB-231) and DOX (12.5 μM for both cell types). The wound closure rate was evaluated using a light microscope set to ×4 magnification in four fields of view. The images were analyzed using ImageJ software (National Institutes of Health, Bethesda, MD, USA), and the migration (closure area) percentages were calculated by the following equation:

$$Migration\;\%=\frac{\left(\text{Area}\;\text{at}\;0\;\text{h}-\text{Area}\;\text{at}\;24\;\text{h}\right)}{\text{Average}\;\text{of}\;\text{area}\;\text{in}\;\text{control}\;\text{cells at 24 h}}\;x\;100$$

### 2.9 Transwell migration assay

MCF7 or MDA-MB-231 (5 × 10^4^) cells were seeded in the upper chamber without FBS. In the bottom chamber of the plate’s wells, 500 μL of complete culture medium (20% FBS) was added. After an incubation period of 6 h, the cells underwent treatment for an additional 24 h. Subsequently, the migrated cells were fixed using 4% formaldehyde in PBS and stained with 0.1% crystal violet for further analysis.

### 2.10 Colony formation assay

A colony formation assay was conducted according to Guzmán et al. (2014), where pretreated MCF7 or MDA-MB-231 cells were plated in 12-well plates at concentrations of 250, 500, and 1,000 cells per well, using 2 mL of medium without drugs. After incubation, which allowed the control wells to develop large colonies, the cells were fixed with 4% formaldehyde in PBS and stained with 0.1% crystal violet for imaging purposes. The color intensity for each group reflects their colony-forming ability, expressed as a percentage of the control. Each well was washed three times with PBS, then eluted with 33% acetic acid to extract the color. The optical density was measured at 570 nm using a spectrophotometric plate reader (Siddiqui et al., 2006).

### 2.11 Reverse transcription-polymerase chain reaction (RT-PCR)

RNA was isolated from cells using the RNeasy Mini Plus Kit (Qiagen NV, Venlo, the Netherlands). Before cDNA conversion, the purity and concentration of the extracted RNA were measured with the iScript cDNA Synthesis Kit (Bio-Rad Laboratories Inc., Hercules, CA, USA). The expression levels of *PKM2* were assessed using quantitative real-time PCR (qRT-PCR) with SYBR Select Master Mix (Thermo Fisher Scientific) on an iQ-5 real-time PCR machine (Bio-Rad), and the results were normalized against *GAPDH* as a housekeeping gene. All primers are listed in Table 1. The PCR setup for this experiment includes the following stages: one cycle at 50 °C for 2 min, followed by one cycle at 95 °C for 2 min. This is followed by 40 cycles, during which the temperature is raised to 95 °C for 2 s for denaturation and then lowered to 60 °C for 30 s for the annealing and extension processes. The fold changes of mRNA expression were calculated by the 2^–ΔΔCt^ method (Livak and Schmittgen, 2001).

**TABLE 1:** Primers’ sequences.

Genes	Sequences	Accession number
*GAPDH*	F: 5-GGATTTGGTCGTATTGGGC-3 R: 5-TGGAAGATGGTGATGGGATT-3	NM_002046.4
*PKM2*	F: 5-CTATCCTCTGGAGGCTGTGC-3 R: 5-GTGGGGTCGCTGGTAATG-3	Designated for this study

### 2.12 PKM2 activity assay

MCF7 or MDA-MB-231 cells were grown in 6-well plates containing 20 × 10^4^ cells until fully confluent. Afterward, the cells received treatments with TA, DOX, and combinations. Cells treated during the glucose uptake assay were harvested for each group and homogenized in RIPA buffer. Following centrifugation, the samples were analyzed for pyruvate kinase activity (Sigma-Aldrich, MO, USA).

### 2.13 Glucose uptake assessment

The culture medium from each group (in pyruvate kinase assay) was collected to assess glucose levels (Sigma-Aldrich, MO, USA) to evaluate the cells’ glucose uptake. The cells were also collected to measure pyruvate kinase activity. Each measurement was made three times (*n* = 3 independent experiments).

### 2.14 Statistical analysis

Statistical analyses were performed using GraphPad Prism 9 (San Diego, CA, USA) with a one-way ANOVA approach, followed by Tukey’s Multiple Comparison Test. IC50 values were determined through nonlinear regression based on the log of the inhibitory concentration with respect to the response variable slope equation. The results are presented as mean ± standard deviation (SD), and a *P*-value of less than 0.05 was deemed statistically significant. All experiments were performed with three independent biological replicates (*n* = 3).

## 3 Results

### 3.1 Bioinformatics assessments

#### 3.1.1 Gene expression analysis of PKM

**FIGURE 1:**
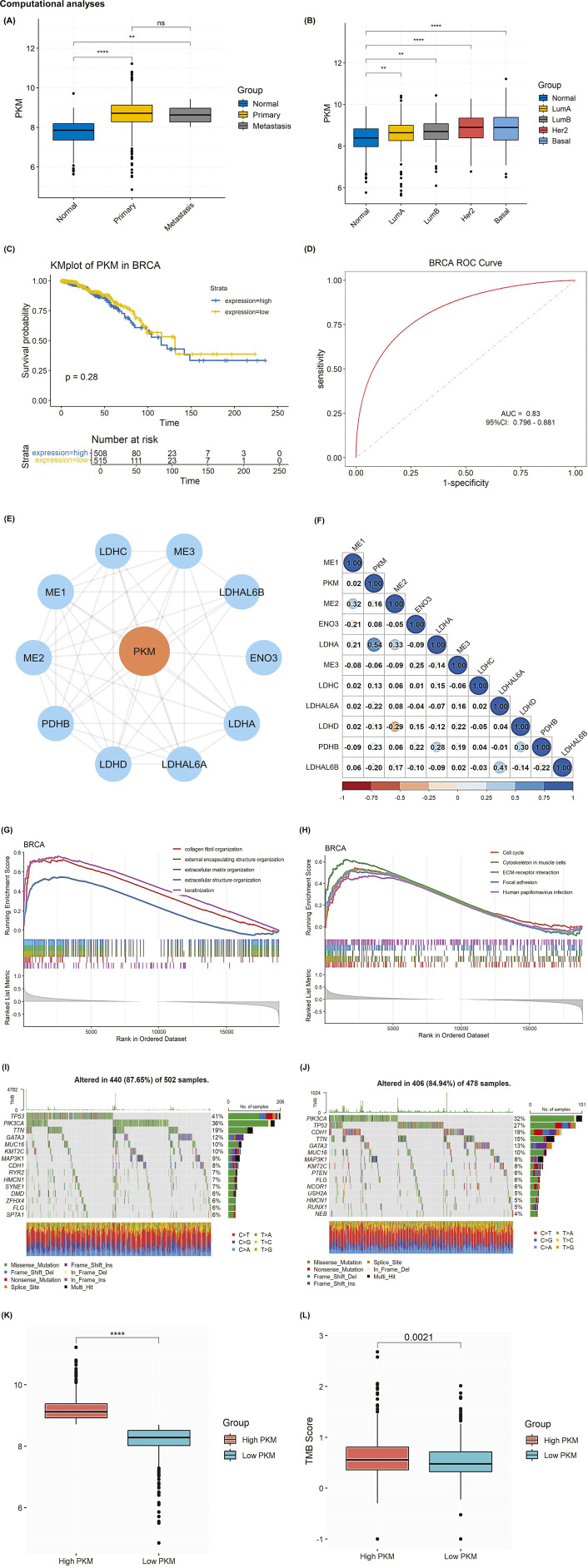
The computational analyses of *PKM* in the breast cancer (BC) dataset **(A)** A barplot illustrating *PKM* expressions across various BC groups (normal, *n* = 113; primary tumor, *n* = 1,106; metastatic, *n* = 7). The metastatic subgroup analysis is limited by the small sample size (*n* = 7), and these results should be considered exploratory **(B)** A barplot demonstrating *PKM* expression in multiple BC subtypes **(C)** A survival analysis of *PKM* in BC tumor samples **(D)** The ROC curve analysis of *PKM* in the BC dataset **(E)** A visualization of the protein-protein interactions (PPI) network of *PKM* extracted using STRING and visualized in Cytoscape **(F)** The correlation among proteins derived from the PPI network **(G)** The Gene Ontology (GO) enrichment analysis of *PKM* in the BC dataset **(H)** The Kyoto Encyclopedia of Genes and Genomes (KEGG) pathway enrichment analysis of *PKM* in the BC dataset **(I)** The oncoplot of the Mutation Annotation Format (MAF) file for the high-*PKM* group **(J)** The oncoplot of the Mutation Annotation Format (MAF) file for the low-*PKM* group **(K)** A barplot comparing *PKM* expression in the high-*PKM* and low-*PKM* groups **(L)** The tumor mutational burden (TMB) score analysis.

The BC transcriptomic data, comprising 1,226 samples, including 113 normal samples, 1,106 primary tumor samples, and only seven metastatic samples, were used to extract the expression of *PKM* and visualized in a bar plot of this gene across the three groups (Figure 1A). *PKM* expressions were upregulated in both the primary tumor group (*P* < 0.001 compared to normal) and the metastatic group, although the latter comparison is limited by the very small sample size (*n* = 7) and should be interpreted with caution; no definitive conclusions can be drawn from this subgroup alone.

The tumor samples are divided into subtypes: luminal A, luminal B, HER2, and basal. *PKM* expression level was upregulated in all subtypes (Figure 1B). Moreover, the prognostic value of *PKM* in BC samples was insignificant, indicating that *PKM* cannot be considered a prognostic marker in BC samples (Figure 1C). Additionally, the Area Under the Curve (AUC) of the ROC curve analysis of *PKM* was 0.83 (Figure 1D).

#### 3.1.2 Protein-protein interaction (PPI) network

The PPI network for PKM revealed ten proteins that directly interact (Figure 1E), including malic enzymes (ME1, ME2, and ME3). Additionally, PKM protein directly interacts with the lactate dehydrogenase (LDH) protein family, including LDHA, LDHAL6A, LDHAL6B, LDHC, and LDHD. Moreover, we calculated the correlation between the PKM protein and the other ten identified proteins. We observed the highest correlation between PKM and LDHA (*r* = 0.54) and the lowest correlation with ME1 (*r* = 0.02) (Figure 1F and Supplementary File 1).

#### 3.1.3 Enrichment analysis

Gene Set Enrichment Analysis (GSEA) was performed using the *TCGAbiolinks* R package to identify biological patterns and pathways in the BC dataset. The collagen fibril organization*,* external encapsulating structure organization*,* extracellular matrix organization*,* extracellular structure organization*,* and keratinization are the top five enriched GO terms (Figure 1G). The cell cycle*,* cytoskeleton in muscle cells*,* ECM-receptor interaction*,* focal adhesion*,* and human papillomavirus infection are the top five enriched in the KEGG pathways (Figure 1H). The complete results of the GO and KEGG pathway enrichment analyses are provided in Supplementary File 2.

#### 3.1.4 Mutational analysis

The median expression of *PKM* in the tumor BC samples was recognized as 8.71. The tumor BC dataset was split based on the median into high-*PKM* and low-*PKM* groups. As a result, 557 samples were classified into the high-*PKM* group and 556 samples into the low-*PKM* group. BC’s MAF file was also downloaded, and the oncoplot was generated for both the high-*PKM* and low-*PKM* groups (Figures 1I,J). The top 15 mutated genes in each group were then visualized. The comparison between the two groups revealed that high-PKM tumor samples had a higher frequency of mutations (87.65% altered in high-PKM vs. 84.94% altered in low-PKM). Moreover, we assessed *PKM* expression in both groups and visualized the results using a bar plot, with a significant outcome (Figure 1K). Finally, we utilized the TMB score to investigate the heterogeneity in BC samples and their association with PKM expression (Figure 1L), yielding a significant result (*P* = 0.0021).

### 3.2 Molecular docking interactions and molecular dynamics (MD) simulations

**FIGURE 2:**
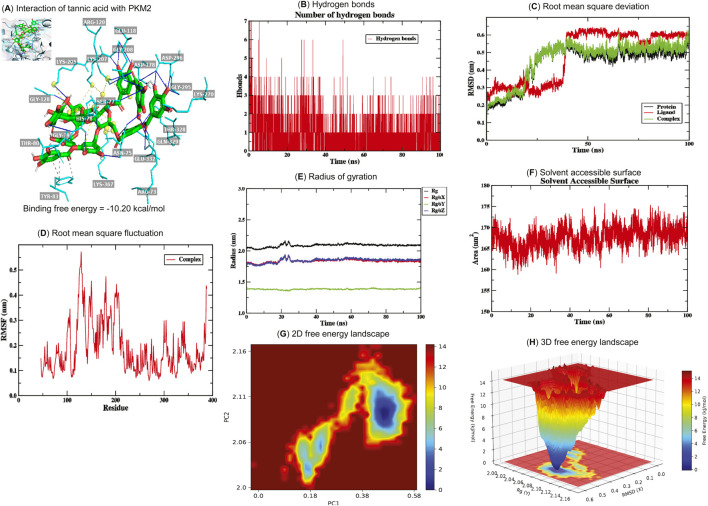
Molecular docking interaction and molecular dynamics (MD) simulations of tannic acid with pyruvate kinase M2 (PKM2) **(A)** Molecular interaction of tannic acid with PKM2 and **(B)** Hydrogen bonds (H-bonds) of MD **(C)** Root mean square deviation (RMSD) of MD **(D)** Root mean square fluctuation (RMSF) of MD **(E)** Radius of gyration (Rg) of MD **(F)** Solvent accessible surface (SASA) of MD **(G)** Two-dimensional (2D) free energy landscape of MD **(H)** Three-dimensional (3D) free energy landscape of MD.

The calculated binding free energies for TA and DOX were −10.20 kcal/mol and −8.80 kcal/mol, respectively, representing a modest difference of 1.4 kcal/mol. Given the typical accuracy range of molecular docking scoring functions (often ± 1–2 kcal/mol). Both compounds showed stable binding conformations in the docking simulations. The molecular docking interactions of TA with the PKM2 protein are represented in Table 2, Figure 2A, and Supplementary Figure 1A. TA interacted with ARG73 (two), ASN75 (three), SER77, HIS78 (two), GLY79 (two), THR80, GLU118, ARG120 (two), GLY128, ASP178, LYS206, GLY208 (three), LYS270, GLY295, ASP296 (four), GLN329, and GLU332 (two) residues by hydrogen bonds, while bound with HIS78, TYR83 (three), and THR328 residues by hydrophobic interaction. In addition, it interacted with HIS78 (five), ARG120 (two), LYS206, LYS207 (three), and LYS367 (two) residues, forming salt bridges.

**TABLE 2:** Molecular interactions of tannic acid and doxorubicin (DOX) with the pyruvate kinase M2 (PKM2) protein.

Ligands	Hydrogen bond	Hydrophobic interaction	Salt bridges	Pi-cation interactions
Tannic acid	ARG73 (two), ASN75 (three), SER77, HIS78 (two), GLY79 (two), THR80, GLU118, ARG120 (two), GLY128, ASP178, LYS206, GLY208 (three), LYS270, GLY295, ASP296 (four), GLN329, and GLU332 (two)	HIS78, TYR83 (three), and THR328	HIS78 (five), ARG120 (two), LYS206, LYS207 (three), and LYS367 (two)	-
DOX	ARG73, ASN75, ASP177, ASP178 (two), GLY208, LYS270, and LYS367	ASP178, THR328, and ALA366	-	HIS78

The complex of PKM2 and TA was able to form an average of 2.8 ± 0.4 stable hydrogen bonds from 4 ns onward in the 100 ns of MD simulation (Figure 2B). The average RMSD for the protein backbone over the equilibrated part of the simulation (from 20 to 100 ns) was 0.23 ± 0.04 nm, with the distribution histogram for RMSD showing a unimodal Gaussian distribution with a 95% confidence interval of 0.21–0.25 nm, indicating a single stable conformational ensemble with no large-scale transition. RMSD of the ligand was 0.38 ± 0.07 nm, 95% confidence interval: 0.34–0.42 nm (Figure 2C). To ensure structural convergence, a clustering analysis using a cutoff of RMSD of 0.1 nm was performed, which found 4 major clusters that accounted for 87% of the trajectory frames, with the largest cluster representing 52% of the frames. The rigid cores (0.12 ± 0.03 nm, backbone fluctuation in the majority of residues) and flexible regions (up to 0.31 ± 0.05 nm, backbone fluctuation in many residues) were mainly found in the loop regions away from the binding site (Figure 2D). The values of Rg were maintained around 2.07 ± 0.03 nm (mean ± SD) (Figure 2E) and SASA around 170 ± 4 mm^2^ (Figure 2F) throughout simulation, suggesting that the compactness of the system was preserved. For the energy–minimized structures, free energy landscapes revealed only one deep minimum and a wide low-energy basin, which was consistent with a stable conformational ensemble (Figures 2G,H).

**FIGURE 3:**
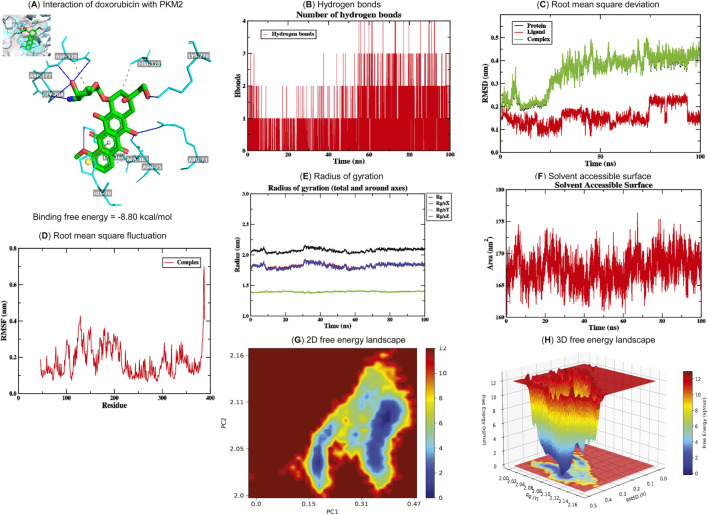
Molecular docking interaction and molecular dynamics (MD) simulations of doxorubicin (DOX) with pyruvate kinase M2 (PKM2) **(A)** Molecular interaction of DOX with PKM2 and **(B)** Hydrogen bonds (H-bonds) of MD **(C)** Root mean square deviation (RMSD) of MD **(D)** Root mean square fluctuation (RMSF) of MD **(E)** Radius of gyration (Rg) of MD **(F)** Solvent accessible surface (SASA) of MD **(G)** Two-dimensional (2D) free energy landscape of MD **(H)** Three-dimensional (3D) free energy landscape of MD.

DOX interacted with the binding site of PKM2 by a binding energy of −8.80 kcal/mol through forming hydrogen bonds with ARG73, ASN75, ASP177, ASP178 (two), GLY208, LYS270, and LYS367 residues, while interacting with ASP178, THR328, and ALA366 residues by hydrophobic interactions and forming pi-cation interaction with HIS78 residue (Table 2; Figure 3A; Supplementary Figure 1B). The interactions between PKM2 and DOX in MD simulations showed a significant increase in hydrogen bonds from 1.9 ± 0.5 in the first 50 ns to 3.1 ± 0.4 in the final 50 ns, suggesting progressive stabilization of the complex (Figure 3B). The protein backbone RMSD is constant at 0.18 ± 0.02 nm (mean ± SD) during the simulation, and the RMSD of the ligand stabilized at 0.11 ± 0.02 nm, while the complex RMSD gradually increased from 0.25 to 0.28 ± 0.01 nm during the simulation (Figure 3C). The RMSD distribution analysis gave 95% confidence intervals of 0.16–0.20 nm for protein and 0.09–0.13 nm for ligand and showed a well-behaved simulation. The three clusters obtained in the clustering analysis accounted for 92% of the trajectory frames, of which 68% belonged to the largest cluster. All but the C-terminal residue (0.65 ± 0.05 nm) were found to be rigid with a mean RMSF of 0.18 ± 0.06 nm (Figure 3D). The Rg varied between 2.00 and 2.14 nm with a mean value of 2.06 ± 0.03 nm (Figure 3E). SASA rapidly increased over the first 10 ns, but then remained more or less constant at 171.5 ± 2.2 mm^2^ (Figure 3F). The 2D free energy landscape contained several low-energy basins, while the 3D free energy landscape (RMSD versus Rg) did not show any other substantial basins, suggesting that DOX shifts and stabilizes the conformation of PKM2 into a well-defined ensemble of structures (Figures 3G,H).

### 3.3 Tannic acid and doxorubicin dose dependently reduce breast cancer cell viability

**FIGURE 4:**
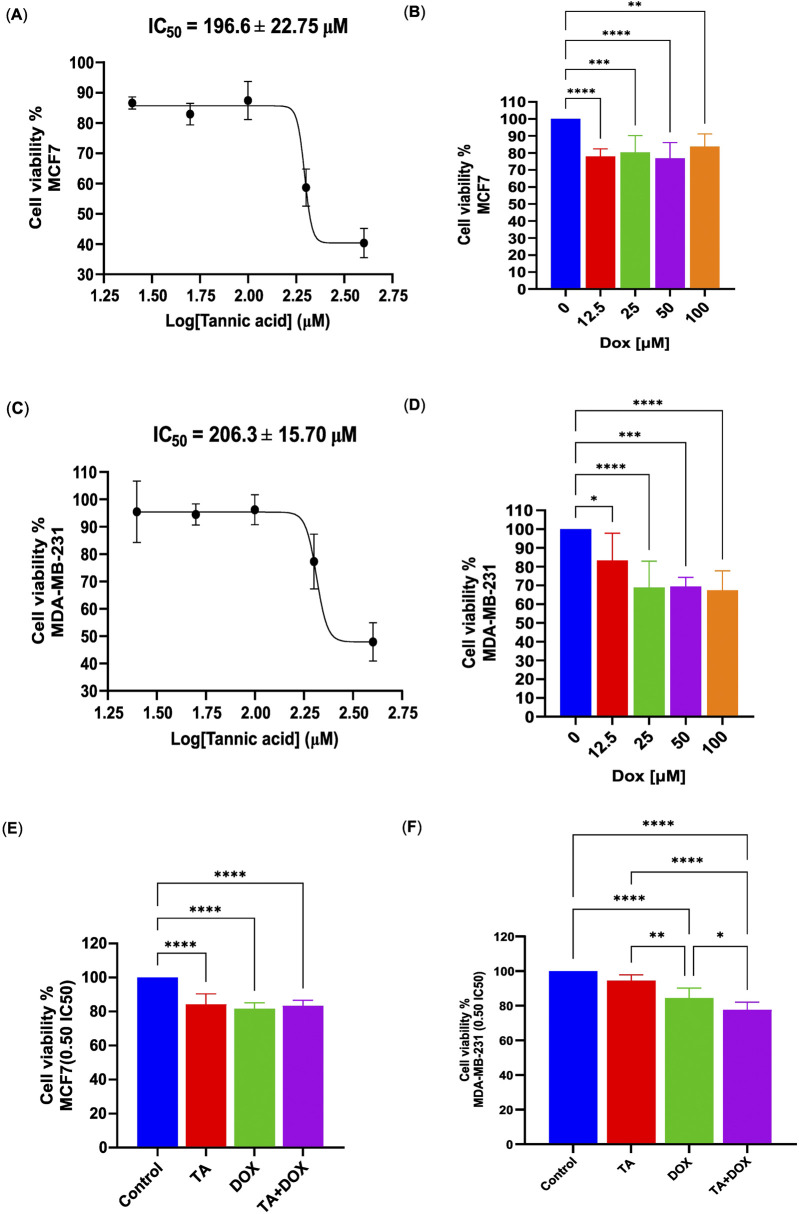
IC50s and cytotoxicity of tannic acid (TA) and doxorubicin (DOX) against MCF7 and MDA-MB-231 cells **(A)** IC50 of TA against MCF7 cells **(B)** Cytotoxicity of DOX against MCF7 cells **(C)** IC50 of TA against MDA-MB-231 cells **(D)** Cytotoxicity of DOX against MDA-MB-231 cells. IC50 values were determined through nonlinear regression based on the log of the inhibitory concentration, concerning the response variable slope equation **(E)** Cytotoxicity of 0.50 IC50 of TA, DOX, and their combinations against MCF7 cells **(F)** Cytotoxicity of 0.50 IC50 of TA, DOX, and their combinations against MDA-MB-231 cells. Data analyzed using a one-way ANOVA approach, followed by Tukey’s Multiple Comparison Test. The results are presented as mean ± standard deviation (SD). ^*^ *P* < 0.05, ^**^ *P* < 0.01, ^***^ *P* < 0.001, and ^****^ *P* < 0.0001. *n* = 3 independent experiments.

The IC50 of TA against MCF7 was 196.6 ± 22.75 μM (Figure 4A), while that against MDA-MB-231 was 206.3 ± 15.70 μM (Figure 4C). Regarding DOX, because its primary role is to induce cell cycle arrest, we used the lowest concentration that induced cytotoxicity in MCF7 and MDA-MB-231 cells (12.5 μM) (Figures 4B–D).

### 3.4 The tannic acid and doxorubicin combination enhances cytotoxicity

A cytotoxicity assay was conducted on MCF7 and MDA-MB-231 cells using concentrations of 0.50 times the IC50. The concentrations at 0.50 IC50 were 98.3 μM for MCF7 and 103.15 μM for MDA-MB-231 for TA, and 12.5 μM for both MCF7 and MDA-MB-231 for DOX. TA (84.25% ± 6.06%), DOX (81.54% ± 3.61%), and TA + DOX (83.33% ± 3.24%) significantly (*P* < 0.0001) increased MCF7 cytotoxicity compared to the control (Figure 4E), while DOX (84.49% ± 5.77%) and TA + DOX (77.63% ± 4.54%) only showed significant (*P* < 0.0001) increases in cytotoxicity in MDA-MB-231 cells compared to the control (Figure 4F). Interestingly, TA + DOX induced more significant cytotoxicity than TA (*P* < 0.0001) and DOX (*P* < 0.05) in MDA-MB-231 cells.

### 3.5 Tannic acid and doxorubicin induce apoptosis, with the combination increasing total cell death

**FIGURE 5:**
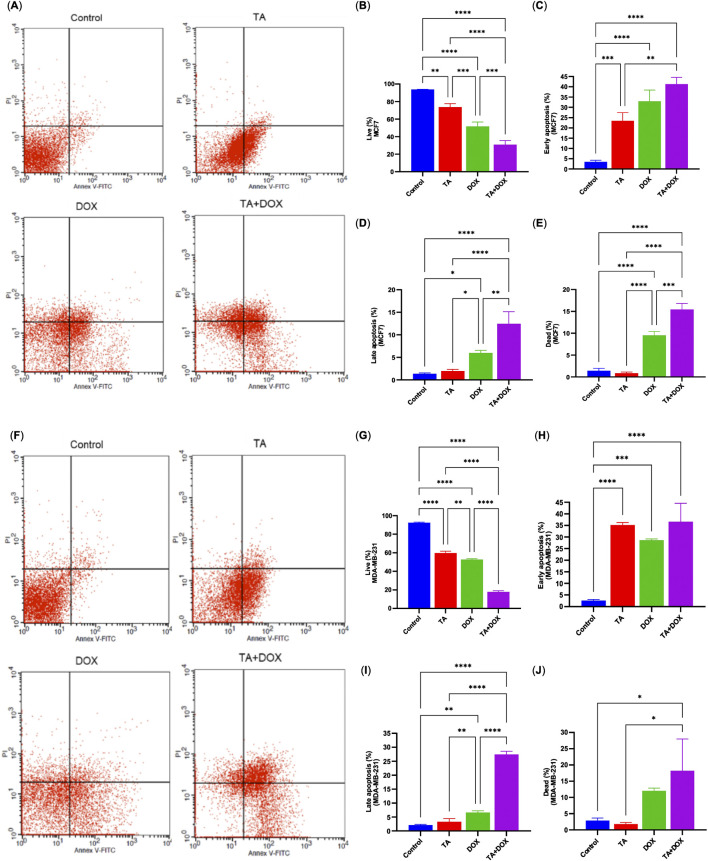
Annexin-V analyses of tannic acid (TA), doxorubicin (DOX), and their combinations against MCF7 and MDA-MB-231 cells **(A)** Flow cytometric analysis of TA, DOX, and their combinations against MCF7 cells **(B)** Live cells percentages of TA, DOX, and their combinations-treated MCF7 cells **(C)** Early apoptosis percentages of TA, DOX, and their combinations-treated MCF7 cells **(D)** Late apoptosis percentages of TA, DOX, and their combinations-treated MCF7 cells **(E)** Dead cells percentages of TA, DOX, and their combinations-treated MCF7 cells **(F)** Flow cytometric analysis of TA, DOX, and their combinations against MDA-MB-231 cells **(G)** Live percentages of TA, DOX, and their combinations-treated MDA-MB-231 cells **(H)** Early apoptosis percentages of TA, DOX, and their combinations-treated MDA-MB-231 cells **(I)** Late apoptosis percentages of TA, DOX, and their combinations-treated MDA-MB-231 cells **(J)** Dead cells percentages of TA, DOX, and their combinations-treated MDA-MB-231 cells. Data analyzed using a one-way ANOVA approach, followed by Tukey’s Multiple Comparison Test. The results are presented as mean ± standard deviation (SD). ^*^ *P* < 0.05, ^**^ *P* < 0.01, ^***^ *P* < 0.001, and ^****^ *P* < 0.0001. *N* = 3 independent experiments.

Flow cytometry results of MCF7 and MDA-MB-231 cells are represented in Figures 5A–E and Figures 5F–J, respectively. Besides, the gating strategies are represented in Supplementary Figures 2A–D and Supplementary Figures 3A–D, respectively. The MCF7 cells treated with TA (*P* < 0.001, 23.45% ± 4.01%), DOX (*P* < 0.0001, 32.95% ± 5.50%), and TA + DOX (*P* < 0.0001, 41.30% ± 3.29%) showed significant increases in early apoptotic percentages compared to the control (3.44% ± 0.79%) (Figure 5C). Additionally, DOX (*P* < 0.05, 5.97% ± 0.58%) and TA + DOX (*P* < 0.0001, 12.45% ± 2.71%) induced late apoptosis compared to the control (1.37% ± 0.17%) (Figure 5D). Similarly, in MDA-MB-231 cells, TA (*P* < 0.0001, 35.22% ± 1.05%), DOX (*P* < 0.001, 28.62% ± 0.56%), and TA + DOX (*P* < 0.0001, 36.63% ± 7.94%) demonstrated significant increases in early apoptotic percentages relative to the control (2.59% ± 0.51%) (Figure 5H). Moreover, DOX (*P* < 0.01, 6.61% ± 0.62%) and TA + DOX (*P* < 0.0001, 27.41% ± 1.17%) resulted in higher percentages of late apoptosis compared to the control (2.12% ± 0.24%) (Figure 5I).

In addition to early and late apoptosis, we calculated total apoptosis (early + late) for both cell lines. As shown in Table 3, total apoptosis in MCF7 cells was significantly increased by TA (25.41% ± 3.95%, *P* < 0.01), DOX (38.92% ± 6.08%, *P* < 0.0001), and TA + DOX (53.75% ± 5.93%, *P* < 0.0001) compared to control (4.81% ± 0.71%). The combination treatment caused significantly more total apoptosis than TA (*P* < 0.001) or DOX (*P* < 0.05) alone. In MDA-MB-231 cells, total apoptosis was markedly elevated by TA (38.53% ± 1.64%, *P* < 0.0001), DOX (35.23% ± 0.07%, *P* < 0.001), and TA + DOX (64.04% ± 8.51%, *P* < 0.0001) relative to control (4.71% ± 0.32%). The total apoptosis caused by the combination was significantly more than that of either TA alone (*P* < 0.001) or DOX (*P* < 0.001) alone. These results indicate that the TA + DOX treatment induces more general cell death than the treatment with either agent alone.

**TABLE 3:** Total apoptosis (early and late apoptosis) in MCF7 and MDA-MB-231.

Cell lines	Control	TA	DOX	TA + DOX
MCF7	4.81 ± 0.71	25.41 ± 3.95^**^	38.92 ± 6.08^****^	53.75 ± 5.93^****^ ^/^ ^+++^ ^/^ ^x^
MDA-MB-231	4.71 ± 0.32	38.53 ± 1.64^****^	35.23 ± 0.07^***^	64.04 ± 8.51^****^ ^/^ ^+++^ ^/^ ^xxx^

Mean ± SD. ^**^ *P* < 0.01, ^***^ *P* < 0.001, and^****^ *P* < 0.0001 vs. control.

^+++^ *P* < 0.001 vs. TA. ^x^ *P* < 0.05 and ^xxx^ *P* < 0.001 vs. DOX.

### 3.6 Tannic acid causes G0/G1 arrest, doxorubicin disrupts the cell cycle, and the combination leads to phase-independent apoptosis

**FIGURE 6:**
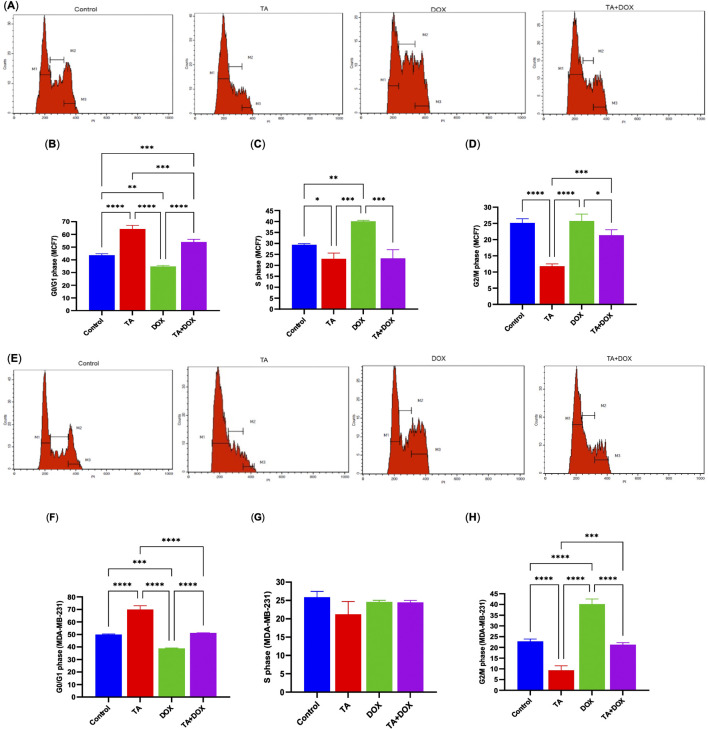
Cell cycle analyses of tannic acid (TA), doxorubicin (DOX), and their combinations against MCF7 and MDA-MB-231 cells **(A)** Cell cycle phases of TA, DOX, and their combinations against MCF7 cells **(B)** G0/G1 phase percentages of TA, DOX, and their combinations-treated MCF7 cells **(C)** S-phase percentages of TA, DOX, and their combinations-treated MCF7 cells **(D)** G2/M phase percentages of TA, DOX, and their combinations-treated MCF7 cells **(E)** Cell cycle phases of TA, DOX, and their combinations against MDA-MB-231 cells **(F)** G0/G1 phase percentages of TA, DOX, and their combinations-treated MDA-MB-231 cells **(G)** S-phase percentages of TA, DOX, and their combinations-treated MDA-MB-231 cells **(H)** G2/M phase percentages of TA, DOX, and their combinations-treated MDA-MB-231 cells. Data analyzed using a one-way ANOVA approach, followed by Tukey’s Multiple Comparison Test. The results are presented as mean ± standard deviation (SD). ^*^ *P* < 0.05, ^**^ *P* < 0.01, ^***^ *P* < 0.001, and ^****^ *P* < 0.0001. *N* = 3 independent experiments.

Cell cycle analysis of MCF7, shown in Figures 6A–D, indicated cell cycle arrest at the G1 checkpoint, where TA showed significant (*P* < 0.0001, 64.23% ± 2.90%) increases in the G0/G1 phase and significantly decreased the S (*P* < 0.05, 23.00% ± 2.64%) and G2/M (*P* < 0.0001, 11.76% ± 0.72%) phases compared to the control. DOX significantly (*P* < 0.01, 34.75% ± 0.86%) reduced G0/G1, while significantly (*P* < 0.01, 40.13% ± 0.40%) increasing the S phase without affecting the G2/M phases compared to the control. Moreover, the combined treatment (TA + DOX) does not induce arrest in the S or G2/M phases, while significantly (*P* < 0.001, 54.01% ± 2.11%) increasing the percentages in the G0/G1 phase.

Cell cycle analysis of MDA-MB-231 showed a significant (*P* < 0.0001, 69.88% ± 3.09%) increase in G0/G1, no change in S, and a significant (*P* < 0.0001, 9.34% ± 2.07%) decrease in G2/M phases in the TA group compared to the control, indicating G1 phase cell buildup with reduced progression to mitosis. In contrast, DOX significantly (*P* < 0.001, 38.83% ± 0.36%) reduced the number of cells in G0/G1, with no change in S phase, and significantly (*P* < 0.0001, 40.16% ± 2.45%) increased the G2/M phase, suggesting cell cycle disruption. Combinations of TA and DOX did not show significant changes in cell cycle phases compared to control (Figures 6E–H).

### 3.7 Tannic acid, doxorubicin, and their combination inhibit breast cancer cell migration

**FIGURE 7:**
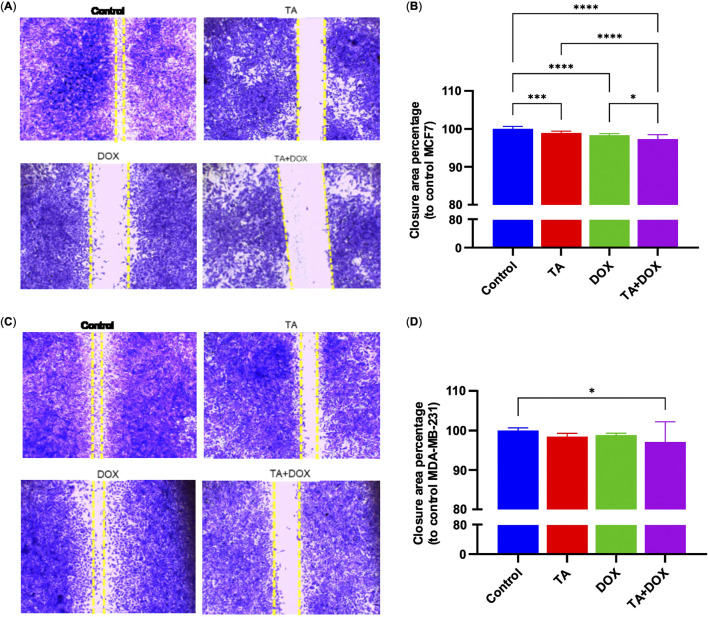
Scratch wound healing assay of tannic acid (TA), doxorubicin (DOX), and their combinations against MCF7 and MDA-MB-231 cells **(A)** Scratch wound healing images of TA, DOX, and their combinations against MCF7 cells **(B)** Percentage of initial wound area remaining open after 24 h (open area %) for MCF7 cells treated with TA, DOX, and TA + DOX. A High percentage indicates poor migration **(C)** Scratch wound healing images of TA, DOX, and their combinations against MDA-MB-231 cells **(D)** Percentage of initial wound area remaining open after 24 h (open area %) for MDA-MB-231 cells treated with TA, DOX, and TA + DOX. A High percentage indicates poor migration. Data analyzed using a one-way ANOVA approach, followed by Tukey’s Multiple Comparison Test. The results are presented as mean ± standard deviation (SD). ^*^ *P* < 0.05, ^***^ *P* < 0.001, and ^****^ *P* < 0.0001. *n* = 3 independent experiments.

MCF7 cells treated with TA (*P* < 0.001), DOX (*P* < 0.0001), and TA + DOX (*P* < 0.0001) exhibited significantly reduced percentages of closure areas in the scratch wound healing assay compared to the control (Figures 7A,B) by percentages of 98.94% ± 0.48%, 98.28% ± 0.49%, and 97.29% ± 1.17%, respectively. The closure area percentages for MDA-MB-231 were significantly (*P* < 0.05) decreased in TA + DOX (97.05% ± 5.16%) compared to the control group (Figures 7C,D). These data demonstrate that TA, DOX, and their combination strongly inhibit cell migration, leaving the wound largely open even after 24 h.

### 3.8 Tannic acid and doxorubicin reduce chemotactic migration across a membrane

**FIGURE 8:**
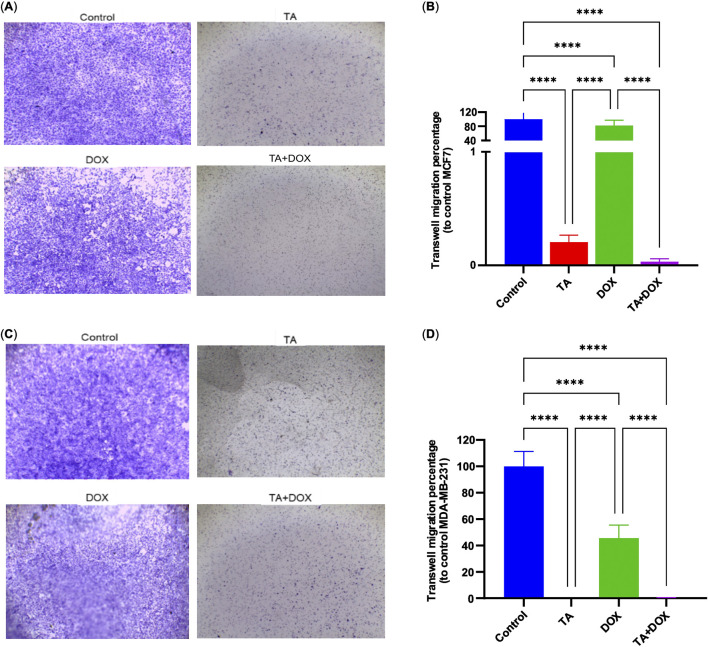
Transwell migration assay of tannic acid (TA), doxorubicin (DOX), and their combinations against MCF7 and MDA-MB-231 cells **(A)** Transwell migration images of TA, DOX, and their combinations against MCF7 cells **(B)** Transwell migration percentages of TA, DOX, and their combinations against MCF7 cells **(C)** Transwell migration images of TA, DOX, and their combinations against MDA-MB-231 cells **(D)** Transwell migration percentages of TA, DOX, and their combinations against MDA-MB-231 cells. Data analyzed using a one-way ANOVA approach, followed by Tukey’s Multiple Comparison Test. The results are presented as mean ± standard deviation (SD). ^****^ *P* < 0.0001. *N* = 3 independent experiments.

The percentages of transwell-migrated MCF7 cells significantly (*P* < 0.0001) decreased in the TA (0.20% ± 0.06%), DOX (82.12% ± 14.90%), and TA + DOX (0.03% ± 0.03%) groups compared to the control (Figures 8A,B). Similarly, compared to the control group, the percentages of transwell-migrated MDA-MB-231 significantly (*P* < 0.0001) decreased in TA (0.02% ± 0.01%), DOX (45.59% ± 9.92%), and TA + DOX (0.33% ± 0.19%) groups, especially the TA-treated group, compared to the control group (Figures 8C,D).

### 3.9 Tannic acid and doxorubicin suppress the clonogenic potential of breast cancer cells

**FIGURE 9:**
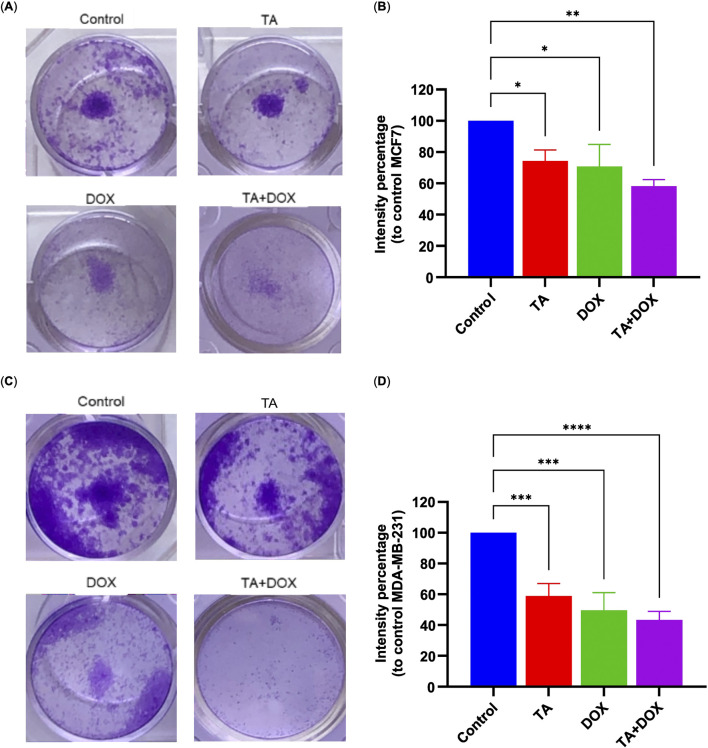
Colony formation assay of tannic acid (TA), doxorubicin (DOX), and their combinations against MCF7 and MDA-MB-231 cells **(A)** Colony formation images of TA, DOX, and their combinations against MCF7 cells **(B)** Intensity percentages of TA, DOX, and their combinations against MCF7 cells **(C)** Colony formation images of TA, DOX, and their combinations against MDA-MB-231 cells **(D)** Intensity percentages of TA, DOX, and their combinations against MDA-MB-231 cells. Data analyzed using a one-way ANOVA approach, followed by Tukey’s Multiple Comparison Test. The results are presented as mean ± standard deviation (SD). ^*^ *P* < 0.05, ^**^ *P* < 0.01, ^***^ *P* < 0.001, and ^****^ *P* < 0.0001. *N* = 3 independent experiments.

The colony-forming abilities of MCF7 cells treated with TA (*P* < 0.05, 74.33% ± 7.04%), DOX (*P* < 0.05, 70.81% ± 14.13%), and TA + DOX (*P* < 0.01, 58.24% ± 4.16%) were significantly reduced compared to the control group (Figures 9A,B). In the same context, MDA-MB-231 cells showed significant decreases in colony formation in TA (*P* < 0.001, 59.01% ± 7.92%), DOX (*P* < 0.001, 49.61% ± 11.49%), and TA + DOX (*P* < 0.0001, 43.37% ± 5.62%) relative to the control group (Figures 9C,D).

### 3.10 Tannic acid treatment reduces PKM2 mRNA expression

**FIGURE 10:**
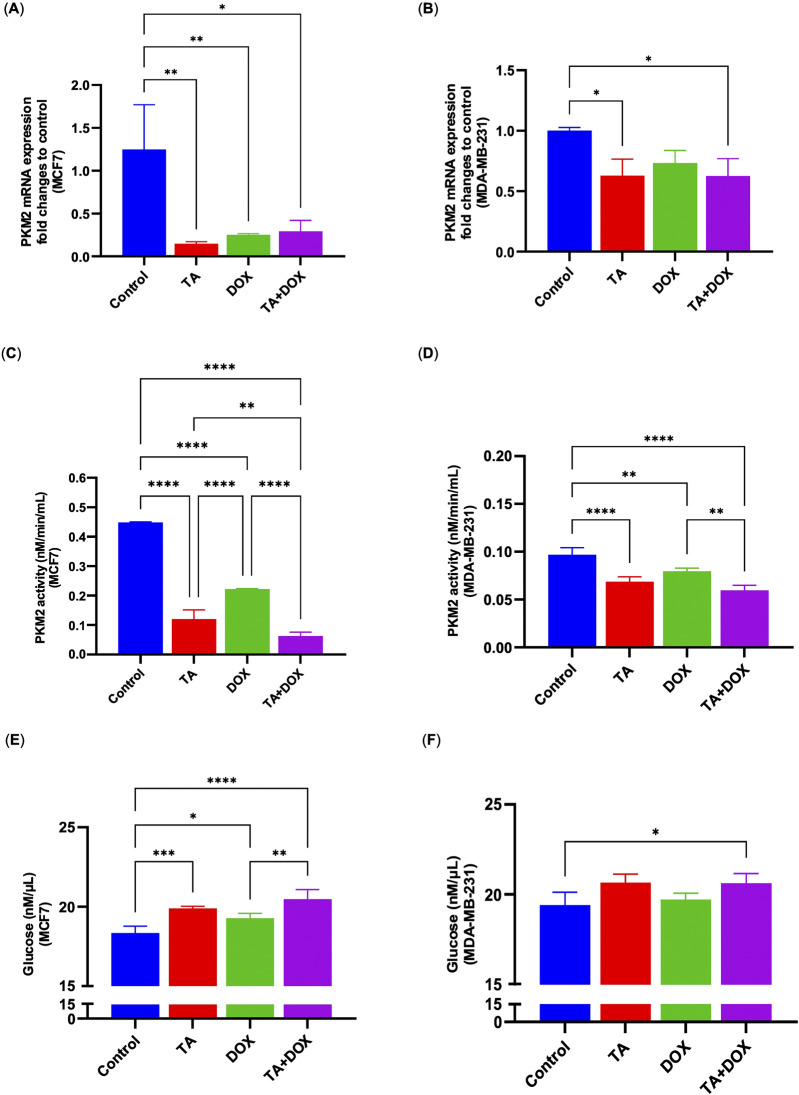
Reverse transcription-polymerase chain reaction (RT-PCR) of pyruvate kinase M2 (*PKM2*), PKM2 activity, and medium glucose levels for MCF7 and MDA-MB-231 cells treated with tannic acid (TA), doxorubicin (DOX), and their combinations **(A)** *PKM2* mRNA expression fold changes of TA, DOX, and their combinations against MCF7 cells **(B)** *PKM2* mRNA expression fold changes of TA, DOX, and their combinations against MDA-MB-231 cells **(C)** PKM2 activity of TA, DOX, and their combinations against MCF7 cells **(D)** PKM2 activity of TA, DOX, and their combinations against MDA-MB-231 cells **(E)** Glucose levels of TA, DOX, and their combinations against MCF7 cells **(F)** Glucose levels of TA, DOX, and their combinations against MDA-MB-231 cells. Data analyzed using a one-way ANOVA approach, followed by Tukey’s Multiple Comparison Test. The results are presented as mean ± standard deviation (SD). ^*^ *P* < 0.05, ^**^ *P* < 0.01, ^***^ *P* < 0.001, and ^****^ *P* < 0.0001. *N* = 3 independent experiments.

The mRNA expression levels of *PKM2* in MCF7 cells were significantly decreased in TA (*P* < 0.01, 0.15% ± 0.03%), DOX (*P* < 0.01, 0.25% ± 0.02%), and TA + DOX (*P* < 0.05, 0.29% ± 0.13%) groups compared to the control (Figure 10A). Additionally, compared to the control group, MDA-MB-231 cells treated with TA (*P* < 0.05, 0.63% ± 0.14%) and TA + DOX (*P* < 0.05, 0.63% ± 0.14%) showed significant decreases in PKM2 expression (Figure 10B).

### 3.11 Tannic acid and doxorubicin inhibit PKM2 enzymatic activity

PKM2 activity was significantly (*P* < 0.0001) decreased in TA (0.12 ± 0.03), DOX (0.22 ± 0.002), and TA + DOX (0.06 ± 0.01) treated MCF7 cells (Figure 10C) compared to control (0.45 ± 0.002 nM/min/mL). Similarly, MDA-MB-231 cells treated with TA (*P* < 0.0001, 0.07 ± 0.005), DOX (*P* < 0.01, 0.08 ± 0.003), and TA + DOX (*P* < 0.0001, 0.06 ± 0.005) exhibited significant decreases in PKM2 activity compared to the control (0.10 ± 0.007) group (Figure 10D).

### 3.12 Tannic acid and doxorubicin reduce glucose uptake

Glucose levels were significantly increased in the medium of TA (*P* < 0.001, 19.88 ± 0.15), DOX (*P* < 0.05, 19.27 ± 0.32), and TA + DOX (*P* < 0.0001, 20.47 ± 0.61) treated MCF7 cells compared to the control (18.34 ± 0.43) group (Figure 10E). At the same time, MDA-MB-231 cells in TA + DOX (*P* < 0.05, 20.61 ± 0.55) exhibited a significant increase in glucose level compared to the control (19.40 ± 0.71) (Figure 10F).

## 4 Discussion

PKM showed good diagnostic potential (AUC = 0.83) but did not have prognostic value for overall survival in BC patients. This contradiction can be explained by the different biological questions that diagnostic and prognostic biomarkers address. Diagnostic biomarkers identify cancer by differences in expression levels between cancerous and normal tissues; PKM is known to be upregulated in various cancers compared to normal tissues (Yang and Lu, 2015; Xue et al., 2025). In contrast, prognostic biomarkers predict clinical outcomes like survival, which depend on tumor heterogeneity, stage-specific gene expression, and interactions with treatments. Therefore, PKM’s diagnostic value comes from its consistent upregulation in BC, while its lack of prognostic significance underscores the complex metabolic changes during disease progression. Further studies using independent cohorts and protein-level analysis are needed to clarify this distinction.

Furthermore, PKM interacts directly with ten proteins, including malic enzymes (ME1, ME2, and ME3), which play vital roles in metabolic processes. Notably, ME1 helps in the production of lipids and cholesterol by aiding in the formation of cytosolic NADPH (Simmen et al., 2020), ME2 drives energy production in the mitochondria (Chen et al., 2023), and ME3 maintains mitochondrial NADPH stability for redox balance (Choudhury, 2021). The PKM protein directly interacts with several LDH family members, including LDHA, LDH-L6A, LDH-L6B, LDH-C, and LDH-D. Among these, LDHA shows a high correlation in this interaction, a finding supported by the research conducted by Wiese et al. (2021), who identified oxaloacetate as a competitive inhibitor of LDHA. They reported that increased PKM2 activity enhances the *de novo* synthesis of oxaloacetate through glutaminolysis, ultimately inhibiting LDHA in cancer cells.

The enrichment analysis highlighted the essential functions of PKM, which include the organization of collagen fibrils and the extracellular matrix, keratinization, regulation of the cell cycle, and interactions with the cytoskeleton, ECM receptors, and focal adhesions. These computational results agree with the experimental studies of Han et al. (2021), Magadum et al. (2020), Lv et al. (2024), and Gao et al. (2022). Furthermore, the biological relevance of enriched human papillomavirus infection (HPV) in BC has been recognized in the current study. Multiple meta-analyses have demonstrated a significant association between HPV and BC risk; HPV prevalence in BC patients was 21.95% compared to 8.96% in controls, with an odds ratio of 3.83 (95% CI: 2.03–7.25, *p* < 0.01) (Karachalios et al., 2023), and the pooled prevalence of HPV in BC tissues was 23% (95% CI: 18%–28%), with HPV-positive individuals showing a 3.6-fold higher risk of developing BC (OR = 3.63, 95% CI: 2.33–5.64) (Koishybayeva et al., 2025). Mechanistically, high-risk HPV E6 protein promotes cell proliferation by stimulating degradation of the tumor suppressor p53 via a trimeric complex comprising E6, p53, and the cellular ubiquitination enzyme E6-AP (Avenhaus et al., 2025). The E7 protein interacts with retinoblastoma (RB) proteins, triggering E2F transcription factor release and enhancing cellular proliferation, while concurrently suppressing cyclin-dependent kinase inhibitors WAF1/p21 and KIP1/p27. Furthermore, the interaction of HPV E6 and E7 with BRCA1 suppresses several BRCA1-mediated tumor suppressor activities, linking HPV oncoproteins directly to a major BC susceptibility pathway (Awan et al., 2023). HPV infection has also been shown to upregulate APOBEC3B, whose synergistic effect with E6/E7 leads to genomic instability, accumulation of cellular mutations, and consequent tumor initiation and progression (Ohba et al., 2014). Additionally, E6 and E7 oncoproteins activate well-known oncogenic pathways, including PI3K/AKT/mTOR and Wnt/β-catenin, both of which are also centrally implicated in breast carcinogenesis (Janiszewska et al., 2025). Taken together, these mechanistic and epidemiological findings justify the biological relevance of the HPV infection pathway enrichment observed in our study.

The comparison between the two groups revealed that high-*PKM* tumor samples had a higher frequency of mutations (87.65%) compared to low-*PKM* samples (84.94%), a difference of 2.71%. But there are a few reasons to believe that there’s something biologically relevant about this comparison. First, the difference in mutation frequency between molecularly stratified groups, within large-scale cancer genome studies, can have important biological implications, especially when combined with differences in the mutational signatures, copy number alterations, or specific driver gene mutations (Alexandrov et al., 2020). However, the biological relevance of the absolute percentage difference is not the only factor; the type, pattern, and functional consequence of mutations matter too. Second, PKM is a major glycolytic enzyme that plays a role in the Warburg effect, and the expression level of this enzyme is associated with TMB and genomic instability through metabolic reprogramming (Israelsen and Vander Heiden, 2015). Changes in cell metabolic pathways due to differential expression of PKM impact the availability of nucleotide precursors and reactive oxygen species (ROS) and subsequently alter the rate and character of somatic mutations that occur in tumor cells (Pavlova and Thompson, 2016). Third, clinical and biological differences between the expression-stratified groups of BC have been well established. For instance, the molecular subtyping of BC is based on mutations that share overlapping rates and are considered as distinct, biologically and clinically, entities in PAM50 (Cancer Genome Atlas Network, 2012). Likewise, pathway analysis, immune infiltration, and outcome data from tumors grouped by metabolic gene expression have revealed substantial differences in pathway activation, immune infiltration, and prognosis in response to subtle shifts of mutation rates (Thorsson et al., 2018). Therefore, while the absolute difference in mutation frequency is modest, the significant association between *PKM* expression and TMB score supports a non-random relationship. Furthermore, high-*PKM* tumors may harbor distinct mutational signatures or specific driver mutations that are not captured by the overall mutation frequency alone.

This might indicate that high *PKM* expression drives a more aggressive tumor phenotype or confers resistance to specific treatments. Additionally, the TMB score examined the heterogeneity of BC samples and their association with *PKM* expression—similarly, Chen et al. (2019) recognized that mutations in the *PKM2* exon-10 region are associated with reduced allosteric activity and increased nuclear translocation, and act as an oncogene-inducing cancer progression. The median *PKM* expression value of 8.71 served as the cutoff to distinguish high- and low-*PKM* groups. This median-based method is common and unbiased in transcriptomic research, as it divides the cohort into two nearly equal groups (557 and 556 samples), reduces the impact of outliers, and is extensively used in TCGA BC studies (Györffy et al., 2010).

The strong correlation between the expression of *PKM* and TMB that we found in our BC data (*P* = 0.0021) is biologically and clinically significant and is worth further discussion. The major isoform of *PKM2* in cancer cells is a master regulator of the Warburg effect, which promotes aerobic glycolysis and metabolic reprogramming, as described by Xue et al. (2025). The elevated levels of ROS and imbalance of nucleotide precursors, both known to increase somatic mutation rates and genomic instability, are direct contributors to increased TMB in *PKM*-high tumors (Pavlova and Thompson, 2016).

High TMB is an emerging biomarker that appears to be predictive of sensitivity to immune checkpoint inhibitors (ICIs) and has been found to be more strongly correlated with response to PD-1/PD-L1 blockade than PD-L1 expression by immunohistochemistry alone (Chalmers et al., 2017). A large meta-analysis demonstrated that TMB-high groups had significantly improved objective response rates (pooled OR 3.31, 95% CI 2.61–4.19, *P* < 0.001), progression-free survival (pooled HR 0.59, 95% CI 0.49–0.71, *p* < 0.001), and overall survival (pooled HR 0.68, 95% CI 0.53–0.89, *P* = 0.004) in response to ICIs (Wu et al., 2019). The higher TMB in our high-PKM BC group could be used to identify a subset of patients that may be more responsive to immune checkpoint blockade.

The mechanism of connection between the expression of the PKM and TMB is further supported by the axis of the expression of PKM2 and the expression of PD-L1. The binding of PKM2 to the promoter of PD-L1 results in significantly higher expression of PD-L1, and high expression of both synergizes to predict worse survival in tumor cells or in immune cells. Furthermore, it was found that PKM2 regulates PD-L1 expression in M2 macrophages and then reduces the number and activity of CD8^+^ T cells, thus impacting the killing of cancer cells and immune evasion (Chen et al., 2021). Therefore, both a genomic component (higher mutational load) and an immunological component (immune evasion via the PKM2/HIF-1*α*/PD-L1 pathway) exist within the association of PKM and TMB.

In the specific context of BC, TNBC carries higher TMB than other subtypes, and high-TMB cases may derive particular benefit from immune checkpoint blockade, making standardization of TMB as a clinical biomarker of critical importance (O’Meara and Tolaney, 2021). Collectively, these findings position PKM expression as a potential metabolic surrogate for predicting immunotherapy eligibility in BC patients, a hypothesis that warrants prospective validation.

Molecular docking, a pre-experimental screening method, revealed TA’s higher affinity for binding to the active site of PKM2. The difference of 1.4 kcal/mol is modest and falls within the typical error range of docking scoring functions. Both compounds show comparable predicted binding; therefore, we investigated their combined effect on PKM2 using biological assays. Furthermore, MD assessments, both PKM2–TA and PKM2–DOX complexes, maintain compact conformations without unfolding, as evidenced by stable Rg, SASA, and free energy minima. TA forms a stable hydrogen-bond network despite gradual ligand RMSD drift, while DOX relies primarily on hydrophobic and Van der Waals interactions with transient hydrogen bonds and adaptive flexibility. Overall, both complexes exhibit tight, persistent binding and local stability, supporting sustained inhibitory activity against PKM2.

Cytotoxicity assay in the current study revealed that the IC50 of TA is 196.6 ± 22.75 μM for MCF7 and 206.3 ± 15.70 μM for MDA-MB-231 cells. These findings are consistent with published literature, as stated by Aybek et al. (2025), where the IC50 is 103.7 μM for MCF7. This may be due to the TA being a high molecular weight polyphenol (MW ∼1701 g/mol) that exhibits cell-type-dependent antiproliferative activity. Furthermore, the physiological relevance of our IC50 values is reinforced by growing evidence that TA and other high-molecular-weight polyphenols can induce anticancer effects via non-cytotoxic mechanisms at concentrations matching our values. At these levels, significant PKM2 inhibition, decreased glucose uptake, apoptosis induction, and suppression of migration and colony formation were still observed. Besides, there has been growing interest in tannins’ pharmacological effects in various disease treatments in recent years. TA is the most basic hydrolyzable tannin recognized by the FDA as a safe food additive (Jing et al., 2022). Regarding the safety margin of TA, Baldwin and Booth (2022) and Samanta et al. (2004) noted that the LD50 value for oral administration of TA in rats is 2,260 mg/kg body weight.

The mechanism of G0/G1 arrest induced by TA is demonstrated to be the result of metabolic stress due to inhibition of PKM2. Increasing the activity of the p53/p21 pathway in p53-wild type cells like MCF7, and increasing the activity of the G1 checkpoint, are caused by the inhibition of PKM2, which results in decreased glucose uptake and ATP production (Xia et al., 2016). Our study demonstrated a significant increase in the G0/G1 phase, and a corresponding decrease in S and G2/M phases, showing the cells to be robust G1 arrest. G0/G1 cell cycle arrest was also observed in p53-mutant MDA-MB-231 cells, but in this cell line, TA worked through a different mechanism by inhibiting the transcription of cyclin D1, which is required for G1/S transition, by inhibiting STAT3 activity (Darvin et al., 2017). To see whether TA was functioning as a G1-arresting factor downstream of p53, the proportion of cells in G0/G1 phase increased in MDA-MB-231 cells, while the number of cells in G2/M phase decreased.

DOX is a topoisomerase II inhibitor that breaks the DNA in a double-stranded manner, which is typically carried out by ATM/ATR-CHK1/2 activation of the G2/M checkpoint (Yang et al., 2010). In MDA-MB-231 cells, DOX produced the expected G2/M accumulation, with increased G2/M and decreased G0/G1 cell percentages. In MCF7 cells, DOX reduced the number of cells in G0/G1 while significantly increasing the S phase without affecting G2/M. This seeming paradox can be explained by the fact that DNA damage induced by DOX activates the checkpoint and induces apoptosis as well. Irreparable damage to cells causes apoptosis to occur directly from G1 or G2 without accumulation in any phase, and results in a net decrease in the number of cells in G0/G1, with no corresponding increase in G2/M, because the damaged cells are eliminated instead of arrested.

The combination (TA + DOX) did not lead to any significant arrest of the cell cycle in either cell line. This does not mean that the effect is not present, but that in bulk phase analysis, the two different arrest mechanisms counteract each other. TA arrests cells in G1 and DOX arrests cells in G2/M (MDA-MB-231) or induces rapid apoptosis (in MCF7). The combination effect of both drugs is a heterologous population of cells arrested at various phases, with no dominant phase accumulation. What is more important is that both drugs caused apoptosis, and the maximal apoptosis rate was achieved in combination. Based on quantitative total apoptosis data, the total apoptosis of the MCF7 cells was found to be significantly increased by TA + DOX compared to TA or DOX. In MDA-MB-231 cells, the combination elevated total apoptosis control, TA, and DOX alone. This means that cells are killed at different stages but do not get “stuck” in one stage. This “phase-independent apoptosis” may be therapeutically useful as it lessens the risk of checkpoint adaptation resistance.

Both ER^+^ MCF7 and triple-negative MDA-MB-231 cells are effectively targeted by TA, where PKM2 mRNA expression and enzymatic activity are significantly decreased in both cell lines when treated with TA alone or together with DOX. In metabolism, the level of glucose in the medium was higher in both lines post-treatment, reflecting decreased glucose uptake, which is consistent with the disruption of the Warburg effect. Furthermore, all the treatments resulted in significant early and late apoptosis. In particular, the highest level of late apoptosis in the MDA-MB-231 cells was observed using TA + DOX compared to DOX alone; the latter is a clinically significant finding due to the poor prognosis of TNBC. Migration and colony formation were inhibited in both cell lines and even more so when both were combined. TA + DOX produced a greater total apoptotic response than either treatment alone in the more PKM2-dependent MDA-MB-231 cells compared to TA or DOX, but was more effective than TA alone in both cases for inhibiting PKM2 and glucose uptake in MCF7 cells. This is congruent with the fact that cells with p53-wild-type cells are equipped with a strong metabolic stress response, mediated by p21-dependent cell cycle arrest, while p53-mutant cells are more dependent on the metabolic stress response provided by PKM2. These results altogether indicate that targeting PKM2 with TA leads to strong effects in both subtypes, and TA + DOX seems to have stronger effects in the aggressive TNBC, thus giving hope that TA can be used clinically as an add-on to DOX chemotherapy, especially in patients with p53 mutant BCs.

The treatment of MDA-MB-231 cells (p53-mutant, TNBC) resulted in a significant increase in total apoptosis compared to TA and DOX. This is clinically important as TNBC is known to be chemoresistant, and p53 mutations have been linked to poor response to traditional chemotherapy (Zhou et al., 2025). We believe this resistance can be overcome by using the combination of TA (a PKM2 inhibitor) and DOX, and our data show that this is indeed possible, most likely by both blocking the enzyme function of PKM2 by TA and damaging DNA by DOX, causing irreversible apoptosis. Total apoptosis of MCF7 cells was slightly lower than that of MDA-MB-231 cells, but both significantly increased when compared to single agents. The higher apparent response in MDA-MB-231 cells is likely due to the increased reliance of more aggressive and metastatic TNBC cells on aerobic glycolysis (Warburg effect) for survival (Pelicano et al., 2006).

The relationship between PKM2 inhibition, cell cycle arrest, and apoptosis is mechanistically interconnected. PKM2 maintains the metabolic fitness required for cell cycle progression through both energy production and nucleotide synthesis. When PKM2 is inhibited by TA, the resulting ATP depletion and nucleotide imbalance trigger the p53-p21 axis in MCF7 cells, leading to G1 arrest. If metabolic stress persists, cells undergo intrinsic apoptosis via Bax/Bak-mediated mitochondrial outer membrane permeabilization (Yamazaki and Galluzzi, 2022). In DOX-treated cells, DNA damage activates p53, which can induce both G1 arrest (via p21) and apoptosis (via PUMA and Bax). The observation that DOX alone decreased G0/G1 in MCF7 without increasing G2/M suggests that p53-wild-type cells efficiently eliminate damaged cells via apoptosis rather than allowing prolonged arrest–a phenomenon known as “mitotic catastrophe” that bypasses classical checkpoint accumulation (Chaikomon et al., 2018; El-Far et al., 2020). In MDA-MB-231 cells, mutant p53 cannot activate G1 arrest, so DOX-treated cells accumulate in G2/M as they attempt to repair DNA damage before entering mitosis. TA, acting independently of p53 via STAT3/cyclin D1, still induces G1 arrest in these cells. The combination removes the G1 block (via DOX) and the G2/M block (via TA), resulting in no dominant phase accumulation–but simultaneously, both drugs activate parallel apoptotic pathways (extrinsic via TA/EGFR/STAT3 or intrinsic via DOX/mitochondrial damage), leading to the highest overall apoptosis. This phase-independent cell death is a hallmark of effective combination therapy and may explain why the combination was particularly potent in the aggressive, therapy-resistant TNBC subtype.

Based on our experimental results and literature data, we propose an integrated mechanistic model to describe the cellular effects (apoptosis, decreased migration, and impaired colony formation) of MCF7 and MDA-MB-231 BC cells in relation to the inhibition of PKM2 by TA and DOX. First, TA directly binds to the PKM2 binding site and thus directly inhibits the activity of PKM2 (Yang et al., 2018). Our molecular docking and MD simulations confirm this direct binding, showing the stability of hydrogen-bond networks and the stability of the complex even after a 100 ns simulation. Second, inhibition of PKM2 enzymatic activity disrupts the Warburg effect–the hallmark metabolic reprogramming of cancer cells whereby glucose is preferentially converted to lactate even in the presence of oxygen (Liberti and Locasale, 2016). Our data confirm this: TA treatment caused a significant decrease in glucose uptake, which was indicated by higher glucose levels in the culture media and lower levels of PKM2 activity. The decreased glucose uptake and impaired glycolysis result in ATP depletion, ROS accumulation, and metabolic stress (Dong et al., 2016; Pavlova and Thompson, 2016). Third, it has non-metabolic effects of PKM2 on apoptosis and cell survival. In the nucleus, PKM2 interacts with and activates critical transcription factors such as STAT3, HIF-1*α*, and *β*-catenin and boosts the expression of survival genes (Yang et al., 2011; Azoitei et al., 2016; Darvin et al., 2017; Gao et al., 2022; Olea-Flores et al., 2024). Specifically, nuclear PKM2 binds to the STAT3 promoter and increases its transcriptional activity, resulting in increased expression of the anti-apoptotic molecule Bcl-2 (Borik and Hussein, 2025). Importantly, TA has been demonstrated to directly inhibit EGFR/STAT1/3 signaling in BC cells, which resulted in a decrease in STAT3 DNA-binding activity, a decrease in expression of Bcl-2, a decrease in localization of Bcl-2 to mitochondria, release of cytochrome c, and activation of the intrinsic caspase-dependent apoptosis pathway (Darvin et al., 2017). Fourth, the observed decrease in the ability of cells to migrate and form colonies after treatment with TA and TA + DOX can be correlated to the mechanism of action of PKM2 inhibition, which involves multiple converging pathways: (i) PKM2 promotes cell migration by upregulating matrix metalloproteinases via STAT3 (Yuan et al., 2024); (ii) PKM2 supplies glycolytic energy to migrating cells and helps maintain cancer stem cells (Kleszcz et al., 2025); and (iii) metabolic stress from inhibition of PKM2 activates AMPK, leading to cell cycle arrest (Markowitz et al., 2024) – this is consistent with the observed G0/G1 arrest in TA-treated cells.

## 5 Conclusion

This study shows that TA and DOX can be used in an effective combination in targeting PKM2 in estrogen receptor-positive (MCF7) and triple-negative (MDA-MB-231) BC cells, and molecular docking establishes that TA has a better binding affinity to PKM2 (−10.20 kcal/mol) than that of DOX (−8.80 kcal/mol). In the experiments, TA treatment significantly decreased PKM2 mRNA expression and enzymatic activity in the two cell lines, effects that were sustained or even amplified upon the addition of DOX, resulting in lower glucose uptake, as indicated by increased glucose levels in the culture media. Functionally, TA and DOX alone and in combination induced a strong response in the induction of apoptosis, effects on cell cycle progression (greater effect with G0/G1 arrest with TA treatment), and inhibition of migratory capacity and colony-forming ability in both cell lines, with the combination displaying a greater cytotoxic effect on both cell lines compared to single agents. The results indicate that TA, as a single drug or in combination with DOX, disturbs glycolytic metabolism of BC cells by inhibiting PKM2, thus limiting energy supply and reducing aggressive cellular behaviors to warrant further research on TA as a complementary therapy to augment the action of DOX in chemotherapy through metabolic vulnerability of BC cells.

## Data Availability

The raw data supporting the conclusions of this article will be made available by the authors, without undue reservation.
