# Supplementary figures and images for: Unveiling the inhibitory effects of tannic acid and doxorubicin combination on pyruvate kinase M2 in breast cancer cells
Source: Front Pharmacol. 2026 Jul 16;17:1818890. doi: 10.3389/fphar.2026.1818890 (PMC13422562; doi:10.3389/fphar.2026.1818890)

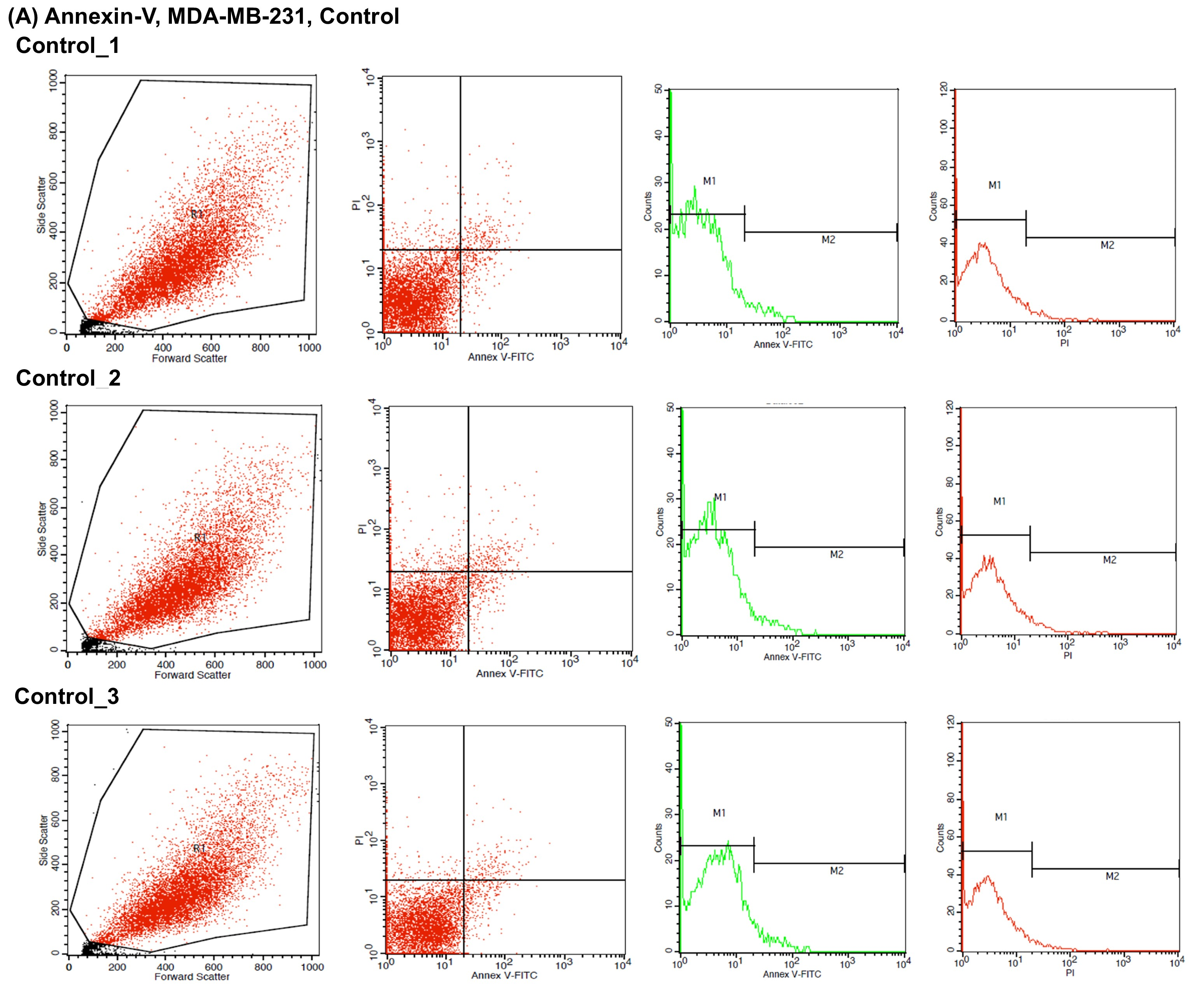

Supplement: Supplementary file 2 [file Image6.tif]

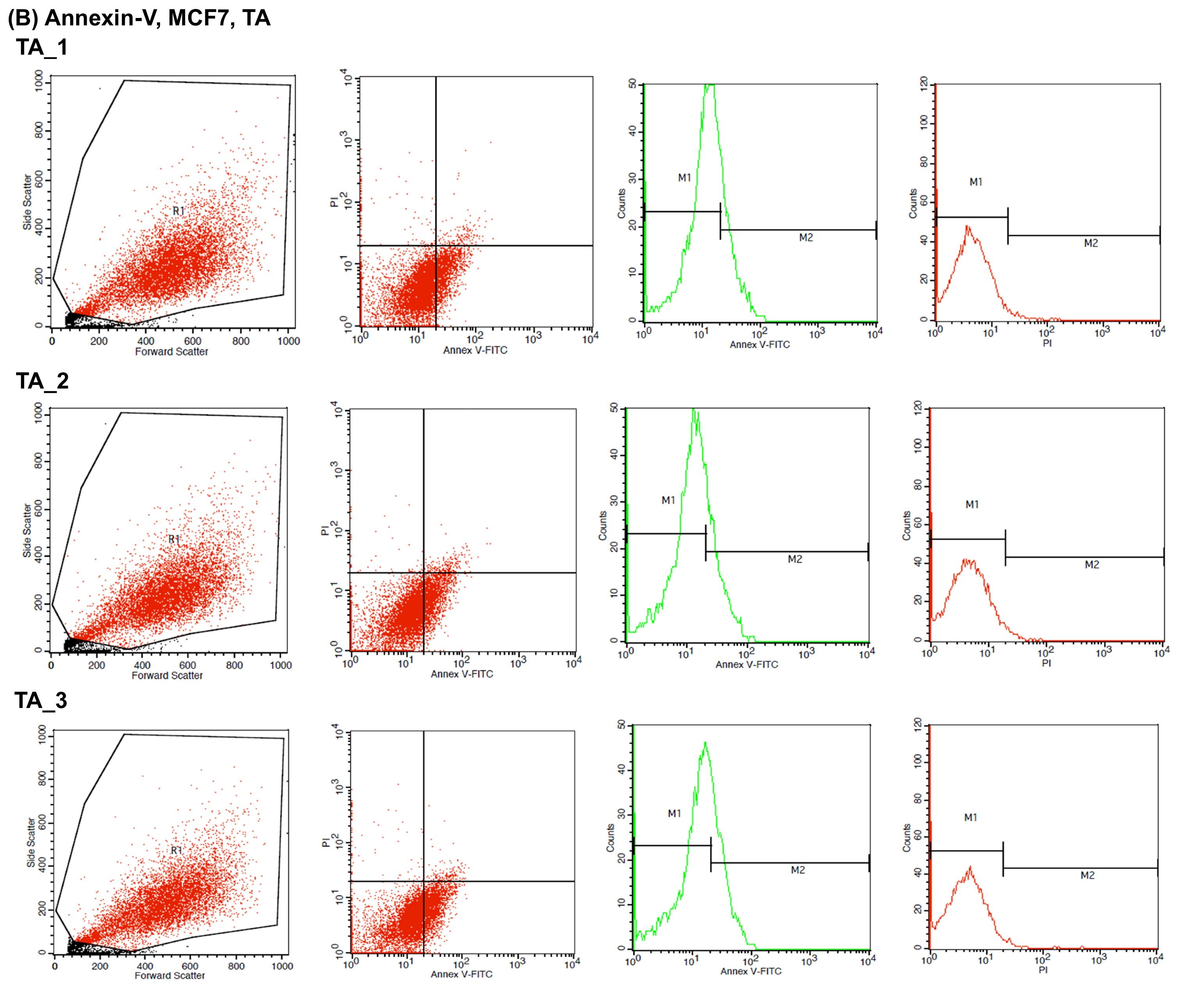

Supplement: Supplementary file 3 [file Image3.tif]

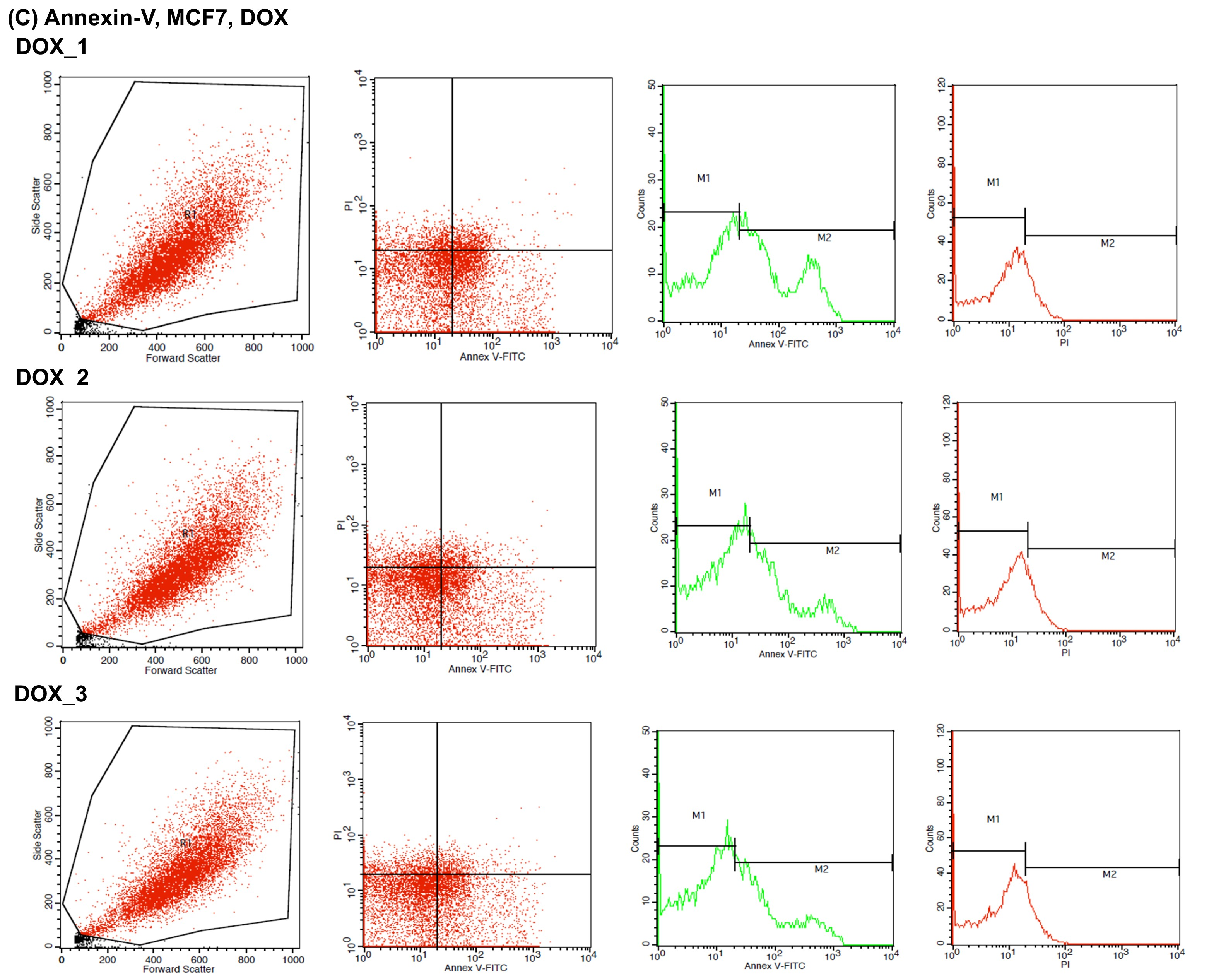

Supplement: Supplementary file 4 [file Image4.tif]

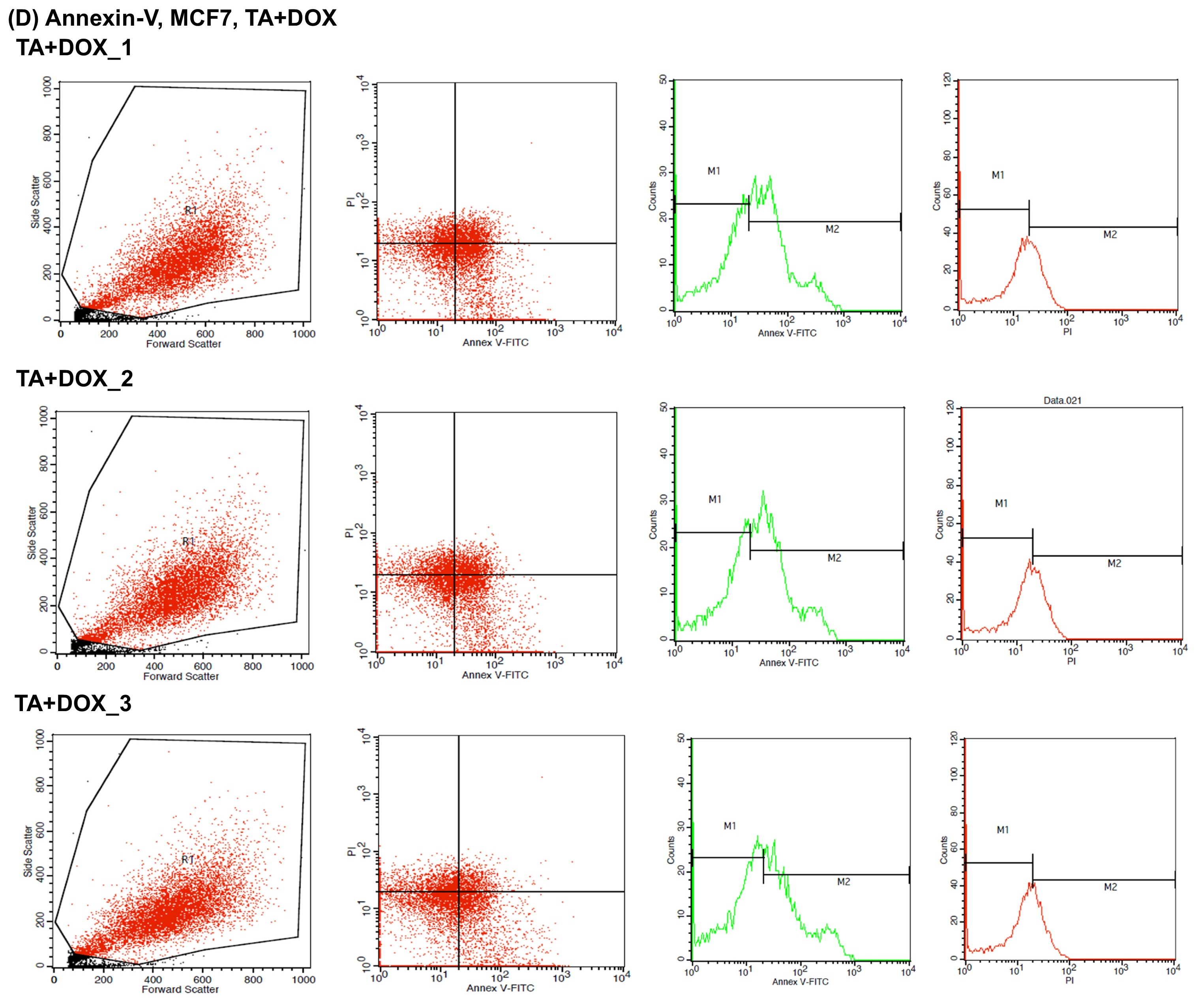

Supplement: Supplementary file 5 [file Image9.tif]

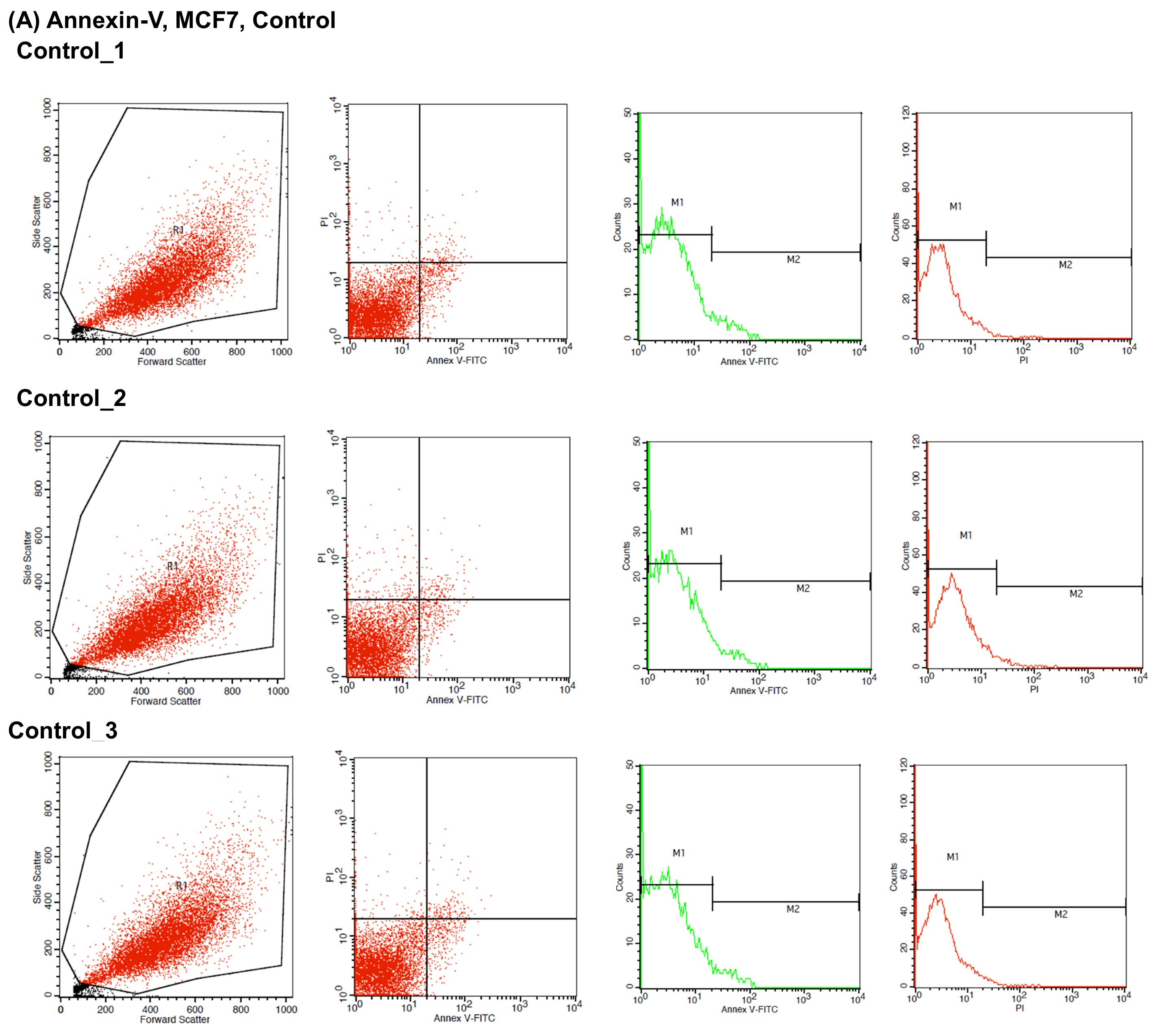

Supplement: Supplementary file 6 [file Image2.tif]

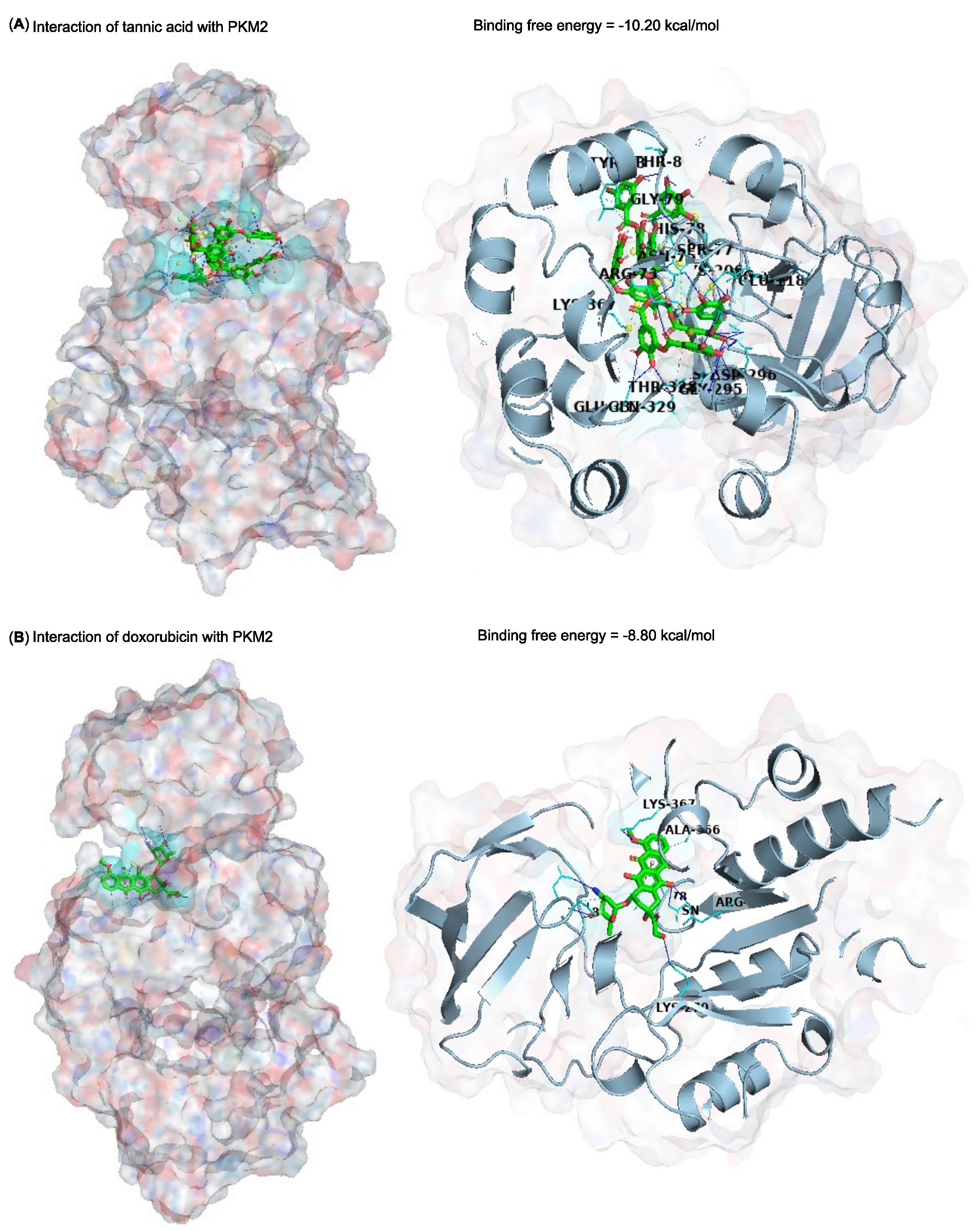

Supplement: Supplementary file 7 [file Image1.tif]

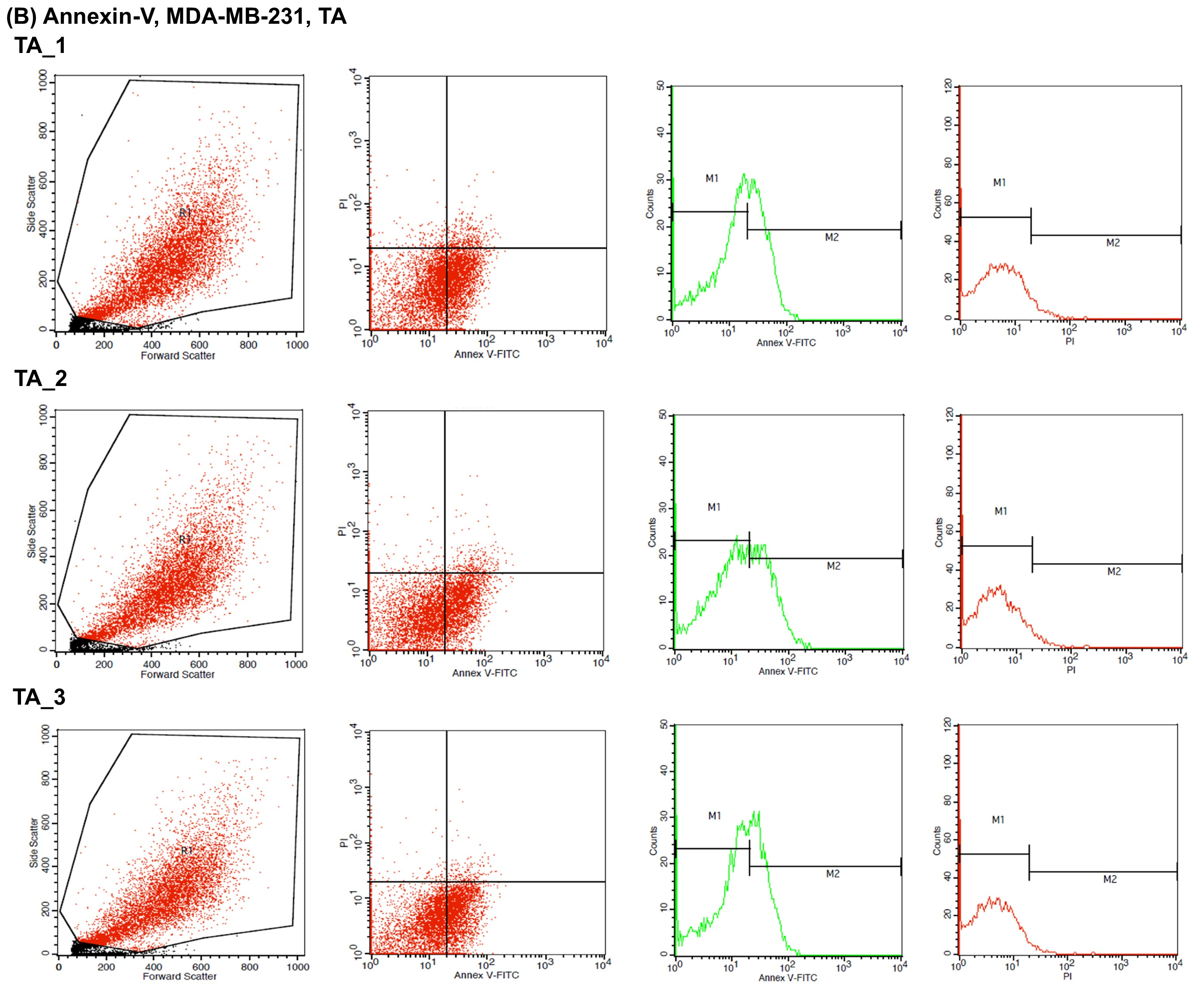

Supplement: Supplementary file 8 [file Image7.tif]

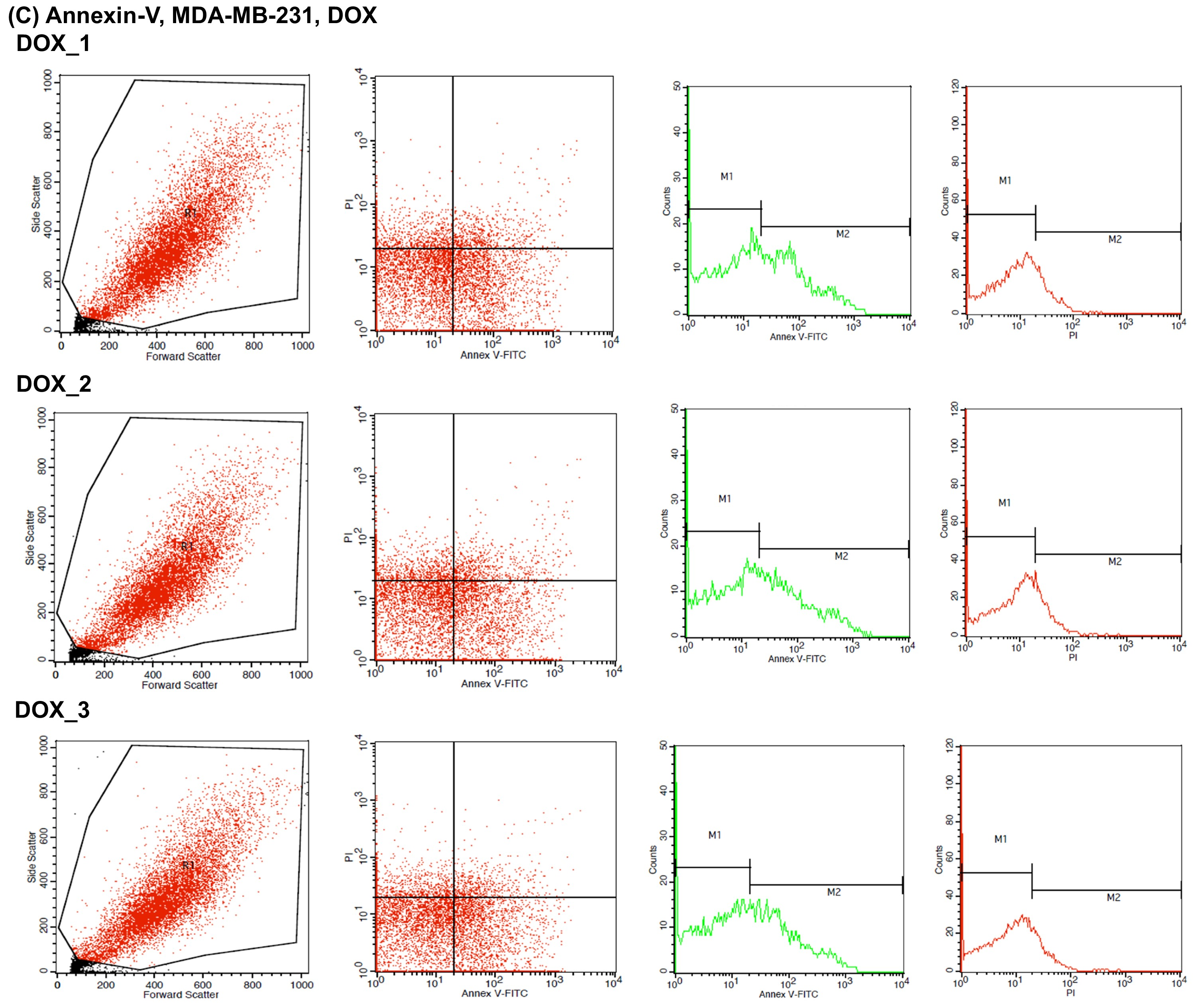

Supplement: Supplementary file 11 [file Image8.tif]

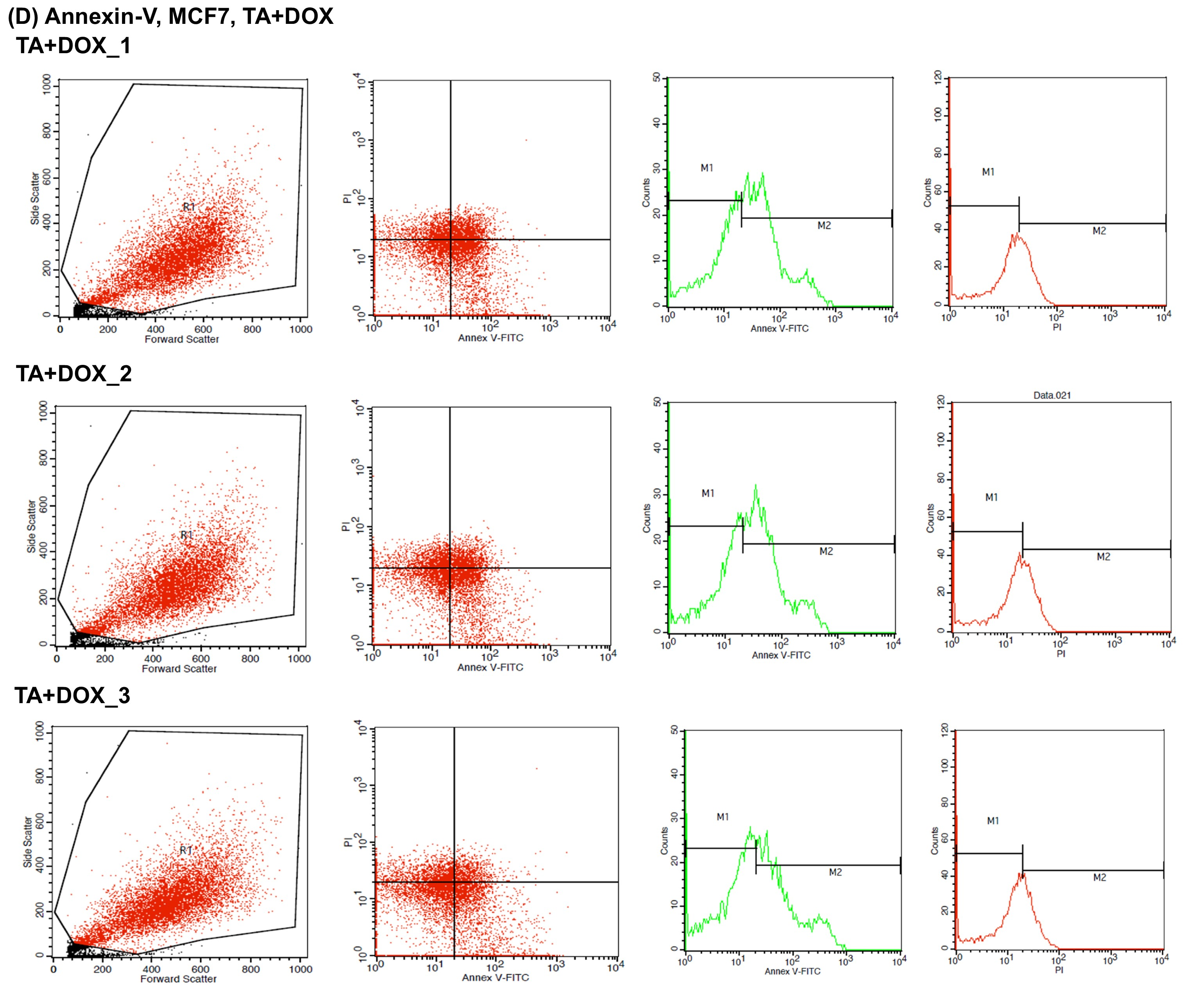

Supplement: Supplementary file 12 [file Image5.tif]
