# Supplementary material for: Unveiling the inhibitory effects of tannic acid and doxorubicin combination on pyruvate kinase M2 in breast cancer cells
Source: Front Pharmacol. 2026 Jul 16;17:1818890. doi: 10.3389/fphar.2026.1818890 (PMC13422562; doi:10.3389/fphar.2026.1818890)
Supplement: Supplementary file 9 [file Table3.docx]

**Supplementary Table 1.** Summary of cellular responses to TA, DOX, and TA+DOX in MCF7 (ER⁺/p53-wild-type) vs. MDA-MB-231 (TNBC/p53-mutant) breast cancer cells, with proposed mechanistic explanations

| Parameters | MCF7  (p53-WT) | MDA-MB-231  (p53-mutant) | Mechanistic explanation |
| --- | --- | --- | --- |
| PKM2 mRNA reduction | TA > DOX > control | TA > TA+DOX > DOX | TA directly binds PKM2; DOX effect indirect via stress |
| PKM2 activity reduction | TA > DOX > control | TA > TA+DOX > DOX | Consistent with mRNA data |
| Glucose uptake (medium glucose) | TA, DOX, TA+DOX increased | TA+DOX increased | PKM2 inhibition reduces glycolytic flux |
| G0/G1 arrest (TA) | Yes (p53/p21 pathway) | Yes (STAT3/cyclin D1 pathway) | Different mechanisms, same outcome |
| G2/M arrest (DOX) | No (rapid apoptosis) | Yes (defective G1 checkpoint) | p53 status determines checkpoint utilization |
| Combination cell cycle | No net arrest | No net arrest | Phase-independent apoptosis |
| Apoptosis (late) | Moderate increase | Strong increase with TA+DOX | TNBC is more dependent on PKM2 for survival |
| Migration inhibition | Strong (all treatments) | Strong (all treatments) | PKM2 supports the metabolic demands of migration |
| Colony formation | Reduced (all treatments) | Reduced (all treatments) | PKM2 is required for clonogenic survival |
| Combination benefit over DOX alone | Modest | Significant | p53-mutant cells are more vulnerable to the metabolic+DNA damage combination |
